# Sexual Health Determinants During the Life Course and Migration of Haitian-Origin People in French Guiana: Protocol for the Parcours d’Haïti Biographical and Transdisciplinary Study

**DOI:** 10.2196/63586

**Published:** 2025-06-12

**Authors:** Leslie Alcouffe, Marc-Alexandre Tareau, Gabriel Brun Rambaud, Aude Lucarelli, Mathilde Boutrou, Camille Thorey, Greta Cantalupi, Karl Kpossou, Florence Huber, Sébastien Rabier, Dévi Rita Rochemont, Théo Blaise, Estelle Thomas, Guerline Jean, Ruth Pierre Louis, Annette Zephirin, Célia Basurko, Félix Djossou, Hawa Cissé, William Faurous, Quentin Drouaud, Mayka Mergeay-Fabre, Antoine Adenis, Mathieu Nacher, Nicolas Vignier

**Affiliations:** 1 Institut Santé des Populations en Amazonie Cayenne French Guiana; 2 Centre d'Investigation Clinique Inserm 1424 Centre hospitalier de Cayenne Cayenne French Guiana; 3 COREVIH Guyane (Regional HIV coordination committee) Centre hospitalier de Cayenne Cayenne French Guiana; 4 Département de Formation et de Recherche en Santé, Université de Guyane Département de Formation et de Recherche en Santé, Université de Guyane Cayenne French Guiana; 5 Institut de Santé Publique, d'Epidémiologie et de Développement, Université de Bordeaux Bordeaux France; 6 Infectious Diseases Department Centre Hospitalier de Cayenne Cayenne French Guiana; 7 Infectious Diseases Department Centre Hospitalier de Kourou Kourou French Guiana; 8 Internal Medicine Department Centre Hospitalier de l’Ouest Guyanais Saint Laurent du Maroni French Guiana; 9 Croix Rouge française de Guyane Cayenne French Guiana; 10 Hôpitaux universitaires Paris Seine-Saint-Denis, Hôpital Avicenne et Jean Verdier Assistance Publique - Hôpitaux de Paris Bobigny France; 11 Inserm Unité Mixte de Recherche 1137, Université Sorbonne Paris Nord, Unité de Formation et de Recherche Santé Médecine et Biologie Humaine Infection, Antimicrobials, Modelling, Evolution (IAME) Bobigny France; 12 Institut Convergences et Migration Aubervilliers France

**Keywords:** HIV, hepatitis B virus, HBV, sexual and reproductive health and rights, transient and migrants, migrants’ health, socioeconomic factors, sexual behavior, violence, gender-based violence, health care quality, access and evaluation, health service accessibility, cultural representations, French Guiana, Haiti

## Abstract

**Background:**

French Guiana, an overseas territory located in the Guiana Shield in South America, faces unique social and demographic challenges, particularly in relation to migration. Haitians represent 1 of the 3 largest foreign communities in the region and face specific barriers to health care access and prevention. They are also a population exposed to HIV infection.

**Objective:**

This Parcours d’Haïti study aims to update knowledge on the health of the Haitian population residing in French Guiana. From a quantitative standpoint, the primary objective of this study was to assess the frequency of sexual behaviors that expose Haitian individuals to HIV risk after arriving in French Guiana and explore the association of this risk with their living conditions. This study adopts a transdisciplinary approach to understand the broader determinants of health and the processes underlying HIV transmission.

**Methods:**

This mixed methods study involves a quantitative epidemiological and biographical analysis of Haitian individuals aged 18 to 60 years who have lived in French Guiana for more than 3 months. The biographical component uses a detailed grid that tracks key life events since birth. The qualitative component includes 3 substudies based on semistructured interviews and focus groups with Haitian people and health care professionals. These aim to deepen understanding of health care access, use of traditional and biomedical care, and mental health among Haitians. A phylogenetic component describes the distribution of clades of the Pol gene in the viruses of people living with HIV. The study materials were cocreated with local stakeholders. Recruitment of this partially hidden population used an innovative method involving all local actors and targeting key locations frequented by the Haitian community. Data collection and recruitment were carried out by Haitian Creole–speaking peer investigators (community health mediators).

**Results:**

Over 20 months between 2021 and 2023, the Parcours d’Haïti study was implemented in French Guiana. Anticipated results suggest that the main reasons for migration include insecurity and fleeing violence. Upon arrival, Haitian people likely experience significant economic hardship and social isolation. Mental health deterioration is expected, with high rates of posttraumatic stress disorder. People living with HIV are expected to face heightened vulnerability compared to HIV-negative individuals even though they already struggle with precarious living conditions. The qualitative findings regarding health care use indicate that Haitian people encounter numerous barriers to accessing health care, such as administrative complexity, language, and financial and mobility issues. The anthropological results are expected to emphasize the magical-religious origins of HIV and the use of plants and traditional medicine for its treatment. Phylogenetic results are anticipated to show a high prevalence of Caribbean B HIV subtypes.

**Conclusions:**

The Parcours d’Haïti study aims to provide valuable insights into the health determinants and HIV-related risks in the Haitian population in French Guiana. These findings should help refine health care policies and practices to better meet the specific needs of this population. Trial Registration: ClinicalTrials.gov NCT05174234; https://clinicaltrials.gov/study/NCT05174234

**International Registered Report Identifier (IRRID):**

DERR1-10.2196/63586

## Introduction

French Guiana is a French South American overseas territory in the Guiana Shield close to the Amazon with a population of 286,618 inhabitants in 2021 [1]. Because of its location and history, this territory is inhabited by people of diverse origins and nationalities [2]. As a result, more than 60 languages are spoken in French Guiana, and approximately one-third of today’s population was born abroad [1-3]. Migration from Haiti represents a major social and demographic challenge in French Guiana [2]. According to 2017 census data, 9.3% of the population in French Guiana originated from Haiti, 1 of the 3 main foreign communities along with those from Brazil and Suriname. It is estimated that 1 in 10 inhabitants speaks Haitian Creole in daily life [3]. Population movements from Haiti have existed since the country’s political turbulence in the 80s and were reinforced by the earthquake in 2010, the closure of the Dominican border in 2015, the 2018 social crisis in Haiti, and the assassination of the Haitian president in 2021. Studies conducted on the sexual health of migrant women in French Guiana in 2021 showed that Haitian women make up the vast majority of the cohorts in health centers set up to welcome migrants, suggesting that migration flows from Haiti continue to be very dynamic [4].

French Guiana is a territory where precariousness is common, with more than half of Guianese inhabitants having a low standard of living and more than 20% having a very low standard of living [5]. This reality particularly affects households where the reference person was born abroad—in that case, more than three-quarters live below the poverty line [5]. After arriving in French Guiana, people’s living conditions deteriorate, not only in terms of housing but also socially and administratively—many people arriving and living in French Guiana do not have a residence permit [4].

French Guiana also faces major health challenges. In 2020, life expectancy at birth was still lower than in continental France in a context of dynamic birth rates [6,7]. In total, 7 major health determinants have been identified by the Haut Conseil de la Santé Publique (High Council for Public Health), including sexual and reproductive health, gender equality, infectious risks, food, nutrition, and mental health [8].

French Guiana is the French territory most affected by HIV, with a prevalence >1% in the general population [9]. Most people living with HIV in French Guiana are of foreign origin [10]. Recent studies in French Guiana (one based on the slope of CD4 decline and the other on virus phylogeny) showed that, among the foreign population living with HIV, more than half had been infected in French Guiana [10,11]. The link between migration and living conditions in the country of arrival has already been established. The Agence Nationale de Recherches sur le Sida et les hépatites virales (the French National Agency for Research on AIDS and Viral Hepatitis; ANRS) Parcours 2012 to 2013 study highlighted the issue of HIV acquisition in the Île-de-France region and late detection of chronic hepatitis B virus (HBV) infections among immigrants from sub-Saharan Africa in relation to their poor living conditions after arriving in France [12,13].

In terms of health and history, Haiti is a country that has been particularly affected by HIV since the 1970s and throughout the epidemic [14]. In 2021, according to the World Bank, 1.8% of the Haitian population was living with HIV [15]. The Haitian population in French Guiana is disproportionately affected by HIV infection—according to surveillance data, 46% of the HIV cohort participants under follow-up in the Cayenne Hospital outpatient department originated from Haiti. Between 2013 and 2018, Haitians represented 36.4% of new HIV infections in French Guiana [16]. Diagnoses are often made later than for native-born people—for Haitian people living with HIV, the time between seroconversion and diagnosis was 4.5 years on average (compared with 3.1 years for people living with HIV born in France) and was 5 years higher among men than among women [17]. The magical-religious importance and impact of stigma has already been explored among the Haitian population in French Guiana [18]. Some diseases, notably HIV, have a high level of social stigma, which can have an impact on people’s use of prevention and care. A similar observation can be made for hepatitis B. In French Guiana, the prevalence of chronic HBV infection is estimated at nearly 1.5%, compared with 0.3% in continental France. Once again, Haitians are particularly affected— the presence of the hepatitis B surface antigen (a marker of hepatitis B infection) concerns 2.5% of Haitian women registered in the French Guiana pregnancy registry [19].

Another study on knowledge, attitudes, and practices showed the high frequency of risky sexual behavior that can expose people to HIV among migrants without, however, specifying them by country of origin. Notably, 35.6% of participants reported engaging in risky behaviors over the previous 12 months (including inconsistent condom use with casual or commercial partners, having multiple sexual partners, or engaging in concurrent partnerships) [20]. A phylogenetic study conducted in French Guiana showed that people from Haiti were less likely to have viruses from large transmission clusters than other immigrants and were more likely to have viruses from small transmission clusters or dyads [11]. Thus, we hypothesize that there are particular conditions leading to a singular epidemiology in this community.

In addition to these local data focusing on sexual health and infectious diseases, international literature highlights several health issues among Haitian migrants worldwide that should be investigated in French Guiana [21,22]. Research suggests that their access to cervical cancer prevention and screening may be inadequate. The mental health of those who have experienced adversity may be impaired, these experiences, along with cultural differences between caregivers and patients, may influence the modalities of care [23,24]. In addition, common health issues could have consequences linked to poorer access to care [25]. The interrelationships among traditional medicine, vodou, herbal medicine, and modern medicine need to be explored [26].

Therefore, all these factors seem to point to the particular needs regarding health, sexual and reproductive health, and rights of the Haitian population, a large and growing part of French Guiana’s population. The Haitian population in French Guiana is a key population for public health in terms of improving not only their health but also the health of the entire Guianese population.

We hereby describe the Parcours d’Haïti study protocol, conceived to update knowledge on the Haitian population living in French Guiana using a transdisciplinary approach to gain a detailed understanding of health determinants and underlying processes of HIV or HBV infection.

## Methods

### Study Objectives

From a quantitative perspective, using notably a biographical approach, the main objective of this study was to evaluate the frequency of sexual behavior exposing people from Haiti to the risk of HIV infection after their arrival in French Guiana and its association with their living conditions. Secondary objectives consisted in describing migration routes, living conditions, access to health insurance coverage and health care, contacts with medico-social structures, social support, general state of health, mental health, and levels of food insecurity after arrival in French Guiana. With regard to sexual and reproductive health and rights, the objectives were to study the delay in first screening for sexually transmitted infections (STIs), as well as the factors associated with earlier screening; study the delay in entry into care and retention in care for people living with HIV or HBV and their determinants; and investigate the use of birth control methods, the frequency of wanted and unwanted pregnancies, and the use of abortion. With regard to emotional life, the objectives were to describe the relational and sexual life.

From a qualitative exploratory perspective, the objectives were to investigate and describe the social representations of HIV and HBV infections and their impact on the use of prevention and biomedical and traditional care. In addition, representations of the Guianese health care system and its prevention services were explored, as well as an anthropological understanding of representations of the body and illness in relation to the ethnomedicinal conceptions of people of Haitian origin living in French Guiana. Finally, another objective was to explore the obstacles to and facilitators of accessing mental health care and understand the mental health problems encountered and the responses provided to migrants in French Guiana from the point of view of health professionals, social workers, and local nongovernmental organizations (NGOs). From a phylogenetic perspective, we aimed to describe the distribution of clades of the Pol gene in the viruses of people living with HIV from Haiti.

Hence, the Parcours d’Haïti study is a mixed methodology study. First, a quantitative component based on an epidemiological (cross-sectional and biographical) study was conducted using a questionnaire and a hetero-administered biographical grid. The questionnaires were administered by Haitian Creole–speaking mediators, for the most part from the local community. Mediators were recruited for their linguistic expertise and interpersonal skills and then trained to collect complex data effectively. Second, a qualitative component based on a socioanthropological ancillary study using participant observation and semistructured interviews (itself developed along several dimensions) was conducted. Third, a phylogenetic analysis of HIV Pol gene sequences based on sequences collected as part of routine care (a resistance genotype analysis is systematically performed before antiretroviral therapy initiation) was conducted. The qualitative component of the research was conducted primarily by a Haitian Creole–speaking anthropologist with extensive experience in studying Creole societies supported by 2 researchers trained in qualitative methodologies.

### Ethical Considerations

This study involved human participants and is covered by French law 2012-300 of 5 March, 2012, known as the Jardé Law, as category 3 research involving the human person, which provides for the analysis of anonymized personal medical data previously collected during routine care. The sponsor and the person directing and supervising the research undertaken ensured that this research was carried out in accordance with the Jardé Law and the Declaration of Helsinki in its latest version.

The study protocol and related participant documents were reviewed and approved by the Comité de Protection des Personnes Sud-Est I (South-East Ethics Committee for the Protection of Persons; October 11, 2021), together with 3 substantial modifications along the course of the study carried out between November 1, 2021, and July 31, 2023. In addition, an ancillary qualitative study has been reviewed and approved by the Comité de Protection des Personnes Sud-Est I (South-East Ethics Committee for the Protection of Persons; May 9, 2022).

The controller and processor of data recorded during this research was the Centre Hospitalier de Cayenne (Cayenne Hospital Center) in accordance with the French Data Protection Act 78-17 of 6 January, 1978, amended by law 2004-801 of 6 August, 2004, and the European Union regulation (EU 2016/679), titled the General Data Protection Regulation.

This research falls within the scope of the Reference Methodology (MR-003). The Centre Hospitalier de Cayenne has signed an undertaking to comply with the Reference Methodology and has a certificate of compliance number 2215826 version 0 from November 20, 2019 (certificate renewed on December 21, 2021, under the number 2224668 version 0). A privacy impact assessment was carried out in accordance with MR-003.

This research was registered on ClinicalTrials.gov under the national clinical trial NCT05174234 and has ID-RCB (National Identifier generated by the French National Medicines Agency) number 2021-A02185-36.

Before the start of recruitment, agreements were signed with all participating centers as well as with relay points to facilitate recruitment by general practitioners, associations, and NGOs.

All those involved in the research have been trained on good clinical practice and the rules of confidentiality and medical secrecy. Before data collection, for all quantitative and qualitative aspects of the study, participants were provided with full information on the research, and a declaration of consent was completed. Finally, each participant was given an anonymized number, and all information was pseudonymized. As a thank you for the time spent in the interview (regardless of which part they participated in), participants received a compensation kit made of toiletry bag containing soap, body lotion, shampoo, condoms, masks, toothbrushes, and toothpaste.

Similarly, semistructured interviews could only be recorded after the participants provided their consent. Concerning viral sequences, after participants’ consent, the Pol gene sequence will be extracted from existing sequences, anonymized, and transferred securely to the partner laboratory at the Oswaldo Cruz Institute of the Oswaldo Cruz Foundation (Fiocruz) in Rio de Janeiro for analysis. No personal or identifying data will be transmitted to Fiocruz.

### Study Populations and Inclusion Criteria

For the quantitative epidemiological and biographical component, participants were included in 3 groups: a general population group, a group of people living with HIV, and a group of people living with HBV.

The inclusion criteria for all groups were as follows: being born in Haiti (regardless of current nationality), having been in French Guiana for >3 months, being aged between 18 and 60 years, and having no objection to participate in the study. For the group of people living with HIV, the inclusion criteria were as follows: living with HIV and having a diagnosis dating back >3 months (whatever their status with regard to hepatitis B and C), as well as being followed up on by one of the study’s partner physicians at the hospitals in Cayenne, Kourou, or Saint-Laurent-du-Maroni; in outpatient care; or by a partner association. For the group of people living with chronic hepatitis B, the inclusion criteria were living with a chronic HBV infection and having been diagnosed for >3 months (not coinfected with HIV), as well as being followed up on by one of the study’s partner physicians in the hospitals of Cayenne, Kourou, or Saint-Laurent-du-Maroni; in outpatient care; or by a partner NGO.

For the general Haitian population group (not known to live with HIV or HBV), the inclusion criteria were declaring that they were not infected with either HIV or HBV, being under the care of one of the study’s partner physicians, or being recruited in the public space of one of the cities targeted by the study (Cayenne, Matoury, Rémire-Montjoly, Macouria, Kourou, Montsinéry-Tonnegrande, Sinnamary, Maripasoula, Saint-Laurent-du-Maroni, or Mana). Exclusion criteria were being unable to answer the interviewer in French or Haitian Creole; being under guardianship, tutelage, or legal protection; and having already answered the study questionnaire.

For the exploratory qualitative sections focusing on health anthropology, mental health, and access to care, the inclusion criteria were tailored to each substudy and comprised the following professionals, including (1) health professionals, social workers, or members of local NGOs with experience working with migrants in French Guiana between 2021 and 2023 who agreed to participate in the study (the mental health and access to care substudies were conducted exclusively with health care professional), and (2) community members, including representatives of the Haitian community who met the inclusion criteria of being born in Haiti, regardless of their current nationality, residing in French Guiana for >3 months and who were aged >18 years and provided consent to participate in the study. For individuals living with HIV or HBV, the same criteria applied, with an additional requirement of being a diagnosed person living with HIV or HBV.

### Sample Size

The number of participants required was calculated to estimate the frequency of the main indicators (sexual behavior that exposes one to the risk of acquiring HIV, including inconsistent condom use with casual or commercial partners, having multiple sexual partners, engaging in concurrent partnerships, and exposure to sexual violence [20]) based on an expected percentage of 50% with a precision of 5% using the following formula:

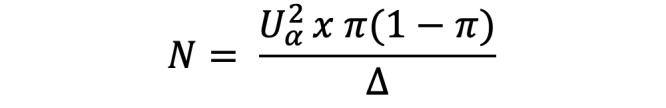



In this formula, 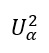 is 1.96 for an agreed risk of 0.05, π is the frequency of the factor studied (in this case, 50% or 0.5), and Δ is the accuracy required (in this case, 5% or 0.05).

From estimates of a total of 384 participants per group, and to take into account incompleteness of data collection, the target was set at 400 people living with HIV and 400 people living with HBV. Considering a 1:2 ratio, 1600 participants were planned in the general population group to have sufficient power to carry out multivariate analyses on all study indicators and conduct analyses on subpopulations of interest, such as women, men, youth, people who arrived after 2015, people living in extreme poverty, and people who have used transactional sex.

Concerning the feasibility of including people living with HIV from Haiti, the number of patients actively being followed up on in Guiana’s hospitals was estimated at 889 in 2021. For the group of people living with HBV from Haiti, the cohort in 2021 was 250 people being actively followed up on in hospital departments. These data are largely underestimated as they do not take into account those who have been lost to follow-up. In terms of the general population, nearly 10% of the Guianese population is of Haitian origin. Thus, the recruitment objectives seem achievable in view of the study’s target population.

### Participant Enrollment, Sampling Approaches, and Monitoring

**Figure 1:**
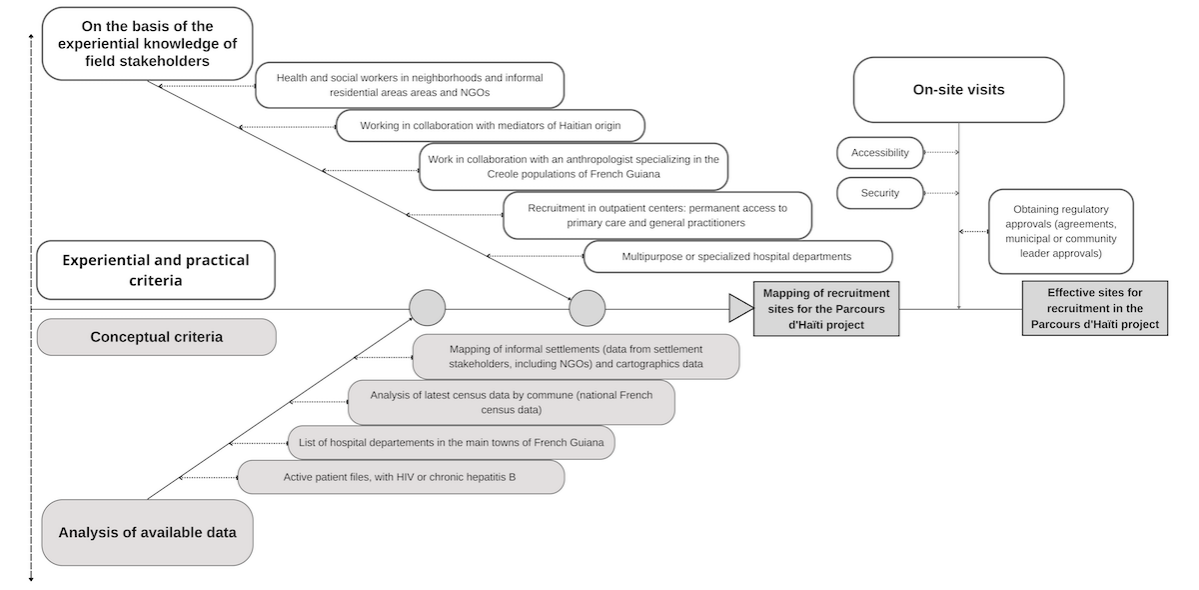
Selection criteria for mapping and choosing locations for recruitment for the Parcours d’Haïti study between 2021 and 2023. NGO: nongovernmental organization.

For the quantitative aspects of the study, sampling of the Haitian population in French Guiana—little explored, partially hidden (living in informal settlements and without papers), and rapidly evolving—was carried out by combining several methods. Recruitment sites were chosen on a nonprobabilistic and reasoned basis. Locations were selected based on literature analysis, census data, knowledge of the peer community workers, knowledge of partner NGOs, and accessibility and safety. These criteria are listed in Figure 1. Finally, partner hospital departments, outpatient centers (health care access offices, French Red Cross prevention and health centers, and primary care centers), and general practitioners were identified. The hospital services selected were those that limited bias on morbidity (particularly chronic diseases). The medical departments selected for participant screening and enrollment were maternity, pediatrics, dermatology, and the emergency room. In view of the enrollment sites mentioned, an oversampling of women was expected and judged as acceptable.

At the recruitment sites, random recruitment was carried out—all people meeting the inclusion criteria were invited to take part in the study. Recruitment was halted once we reached a reasonable number of participants from a statistical power perspective and representativity of the study populations.

To improve the representativeness of the sample, additional recruitment using the snowball method was planned among eligible health and social care professionals and resource persons. Each volunteer participant received 8 cards with an alphanumeric code to be distributed to eligible people within their circle. Those who received a card and wished to participate were able to contact the peer investigators to schedule an interview according to their availability. The entire protocol was explained to them, and they were able to ask any questions they wished before agreeing to take part. This method was used to improve the quality of the sample and minimize selection bias in terms of care and social support as well as insecurity.

For the qualitative components, the samples were chosen based on predefined inclusion criteria, developed previously, to represent the different profiles of stakeholders and individuals. Sampling was stopped for each component when data saturation in the answers was reached.

### Study Monitoring

According to the monitoring plan, the compliance of participants’ nonopposition forms (which are equivalent to informed consent forms—the term “nonopposition” is used under French law for studies conducted under Reference Methodology 003) was verified by a clinical research assistant, along with participants’ identity, inclusion criteria, and inclusion group. If reported by the monitoring, participants duplicates or unmatched inclusion criteria led to participants’ exclusion. In addition, where possible, nonopposition forms were completed by the study investigators in conjunction with the clinical research associate. Before data entry, all information collected on the participants’ biographical grid was proofread daily by a peer investigator. In the event of incomplete or inconsistent data, a review was carried out with the investigator who completed the questionnaire. Finally, the data entered were analyzed and checked for logical correctness.

### Data Collection Tools

All the study’s collection tools were coconstructed with health mediators (community health workers) of Haitian origin, the partner structures, the study’s partner physicians (HBV and HIV specialists), the team from the Regional HIV coordination committee (COREVIH) Guyane, researchers from Inserm Clinical Investigation Center 1424, and the study’s scientific partners. The issues of acceptability, confidentiality, and quality were at the center of all discussions. The data from Haitian-born people aged 18 to 60 years included in the quantitative epidemiological section were collected using 2 tools: a cross-sectional epidemiological questionnaire on a tablet (Multimedia Appendix 1) and a paper biographical grid where information was collected year after year (Multimedia Appendix 2). These tools were pretested, and adjustments were made. These questionnaires were both hetero administered, with interviews being conducted in either French or Haitian Creole, and generally took approximately 60 minutes to complete per participant. The interviews took place at the time they were proposed or during an appointment scheduled at the participants’ convenience.

The cross-sectional epidemiological questionnaire collected data related to arrival and stay in French Guiana, history of administrative procedures to stay for medical reasons, sexual and reproductive health, coerced or transactional relationships over the lifetime, use of birth control methods before and after arrival in French Guiana, use of HIV screening tests, general health status, mental health status (4-item Patient Health Questionnaire scale and the psychotrauma scale of the Post-Traumatic Stress Disorder-8), substance use (Alcohol Use Disorders Identification Test–Consumption scale), health insurance coverage, refusal of care experience, food insecurity and hunger, and use of nonallopathic medicine [27-29]. For the groups of participants living with HIV or HBV, 2 short complementary modules were completed in the cross-sectional questionnaire, follow-up and treatment histories were also collected biographically, and medical questionnaires containing routine biological and medical information were completed (Multimedia Appendix 1). Cross-sectional questionnaire data were collected using the ODK Collect software (Get ODK Inc) on tablets. Transversal data entry was conducted offline, and then data were extracted manually using an R script (R Foundation for Statistical Computing) to transform instances into a CSV database [30]. Biographical data were collected on paper biographical grids and then coded using a dictionary of variables and corresponding labels.

The second main tool used was a biographical grid, with the following information collected retrospectively year after year over the lifetime: residential history, administrative history, health coverage, history of professional activities, sources of income, history of relationships, pregnancies and children, history of screening tests for HIV, HBV and cervical cancer screening, history of illnesses and hospitalizations, health or social support structures encountered since arrival in French Guiana, perceived annual well-being, hunger, and significant events. For the groups of participants living with HIV or HBV, follow-up and treatment histories were also collected biographically. The biographical grid data were then entered into Ennov Clinical by a clinical study technician. The decision to freeze the quantitative databases was taken on March 13, 2024, once the data entry and control processes had been completed. The controls continued until June 7, 2024.

Migration routes were also collected on a map and then entered manually into a CSV database (Multimedia Appendix 3).

For the groups of participants living with HIV or HBV, 2 short medical questionnaires containing routine biological and medical characteristics were completed by the physicians in charge of the patients based on their medical history and the results of biological tests.

**Figure 2:**
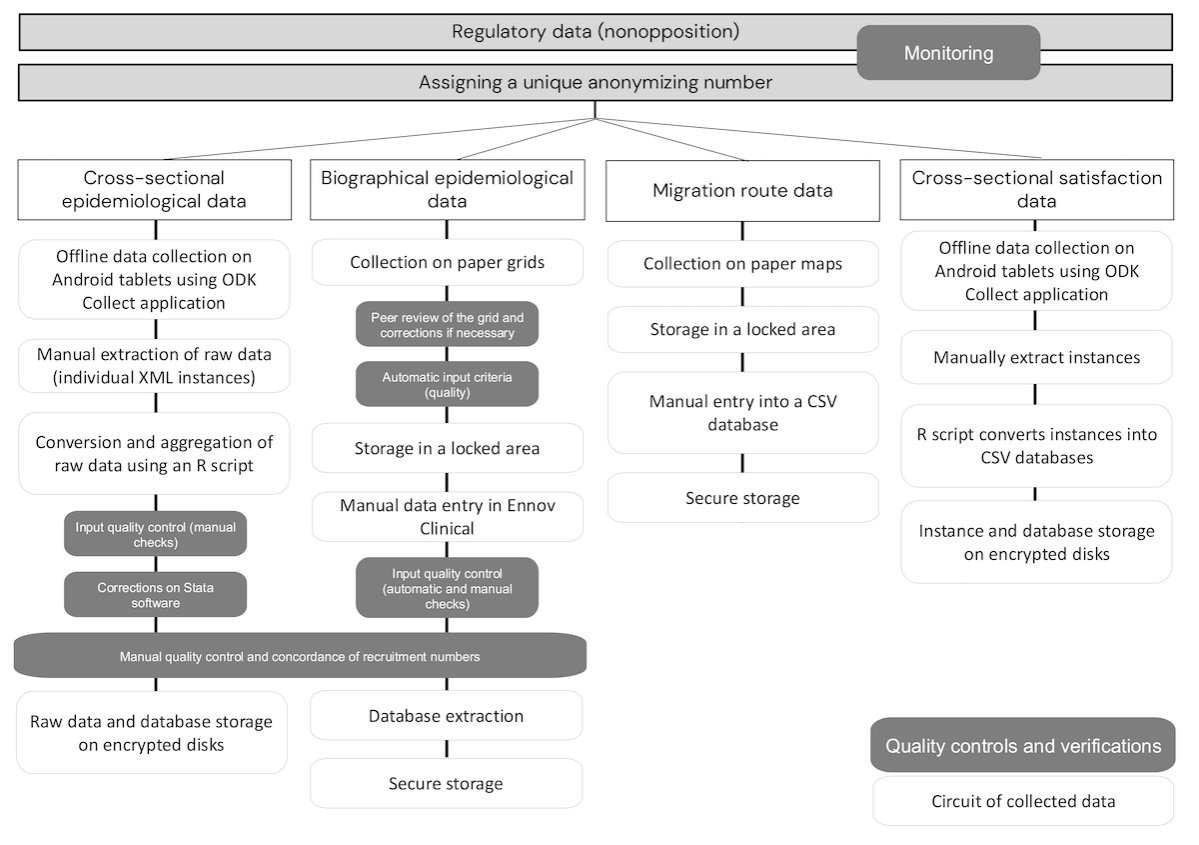
Flow of quantitative, epidemiological, and biographical data collected during the Parcours d’Haïti study and quality control and checks on the data.

Finally, a satisfaction questionnaire was administered via telephone at the end of the study to participants who agreed to leave their contact details with the investigators, focusing on the experience and potential benefits derived from taking part in the study and the in-depth personal interview with a mediator (Multimedia Appendix 4). This section was collected by an investigating mediator via telephone using the ODK Collect software. The flow of quantitative, epidemiological, and biographical data, as well as quality control and data verification processes, are detailed in Figure 2.

Qualitative data were collected via semidirective face-to-face interviews, each lasting approximately 45 minutes (Multimedia Appendix 5). With regard to access to health care, the themes addressed were health determinants and access to and use of medical care by migrants in French Guiana. In the case of mental health, we explored the mental health problems of migrants in French Guiana, existing health care services, obstacles encountered, violence experienced, and its link to sexual health. The interviews were in part recorded using a smartphone, anonymized, and fully transcribed.

An ethnographic dimension was also added to the qualitative axis with active observation in Haitian migrants’ neighborhoods. Traditional practitioners were followed in their care the preparation of traditional remedies, as well as in the treatment of patients whenever possible.

### Analysis Plan

As the analyses had not yet begun at the time of submission of this work, this section is written in the future tense.

#### Statistical Analysis of Quantitative and Biographical Data

With regard to the statistical analysis of quantitative information, the major indicators will be described using percentages as well as means or medians with their SDs or IQRs depending on their distribution. Analytical work will be carried out with a biographical and social epidemiology approach using the tools of epidemiological analysis to study the associations between behaviors and health indicators and social determinants. Factors associated with biographical key indicators will be analyzed year after year using discrete-time logistic regression models that take into account time and the values of indicators collected in the biographical grid year after year [31]. Factors associated with indicators collected at the time of the study will be analyzed using Poisson regression with robust variance. The Stata software (StataCorp) will be used to conduct the analyses.

An analysis of the participants’ support and care structures will be carried out based on specific questions that will be analyzed through clustering and a bipartite network approach using methods derived from graph theory. These analyses will provide a better understanding of the levers and care itineraries that can be developed. The R software will be used for these analyses (*tidyverse* and *tidygraph* packages).

#### Qualitative Analysis

The qualitative study is of a hypothetical-deductive type conducted following a discursive and recursive approach using semidirective interview grids for individual interviews and in a freer manner for discussion groups. These interviews involve a conversational dynamic in which the interviewer and participants interact in a progressive, recursive, and flexible manner to obtain a broad and comprehensive view of the subject. All interview observations will be triangulated with field observations. The collected data (recorded or handwritten) will be analyzed thematically, manually, or using a specialized software (MAXQDA 2022; VERBI GmbH).

#### Phylogenetic Analysis

For the analysis of Pol gene clades, the data (nucleotide sequences) will be extracted from the HIV resistance genotype by the Pasteur Institute laboratory in French Guiana, an analysis conducted for all people living with HIV before antiretroviral therapy. A phylogenetic tree will be produced from these sequences, and the clades to which they belong will be analyzed. The results of the phylogenetic analyses (clades) will be described in the form of phylogenetic trees and cross-referenced with social and biographical determinants, and associations will be analyzed using chi-square tests conducted using the Stata software. These analyses will be carried out in part by the Oswaldo Cruz Institute of Fiocruz in Rio de Janeiro.

## Results

### Overview

For 20 months, the Parcours d’Haïti study was implemented and conducted in French Guiana. The initial study was enriched and developed, and 3 substantial modifications were made.

**Figure 3:**
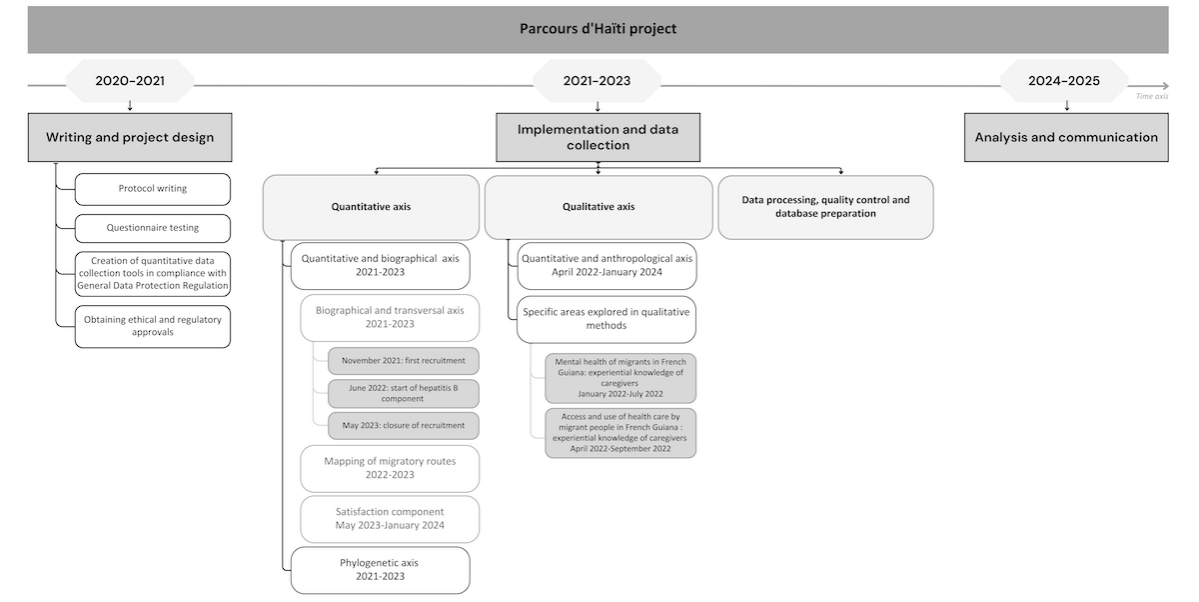
Chronological timeline of the different axes of the Parcours d’Haïti study between 2020 and 2025.

In November 2021, the first participant was recruited for the quantitative epidemiological component, which focused on people living with HIV and the general Haitian population. In June 2022, a group of people living with HBV was added to the study. Several approaches were developed using a mixed methodology: a quantitative cross-sectional approach; a quantitative biographical approach; a qualitative mental health approach; a qualitative approach to access and use of care; an anthropological and ethnobotanical component; and, finally, a “satisfaction” component. These elements are shown chronologically in Figure 3.

### Quantitative and Biographical Data

For the quantitative component, recruitment was completed by May 31, 2023. Data quality control has been extended to June 2024. Analysis is scheduled to begin in July 2024. The collection of migratory routes was completed on May 31, 2023. The first results are expected at the end of 2024.

### Qualitative and Anthropological Data

Qualitative data were collected along the 3 axes mentioned previously. The first section on mental health has been completed, and the first results on mental health are expected at the end of 2024. The section on access to and use of health care has been completed, the interviews have been analyzed, and this work was published in October 2023 [32]. Data collection for the anthropological component ended in January 2024, with the first results expected in September 2024.

### Phylogenetic Analysis

Phylogenetic data have been collected, with the first results expected by the end of 2024.

## Discussion

### Expected Findings

The expected results are described on the basis of findings from the literature. Given recent events in Haiti, most individuals are likely to have migrated within the past 10 years, driven primarily by insecurity and violence. Upon arrival in French Guiana, these individuals are expected to face significant economic hardship and social isolation, which may increase the likelihood of transactional sexual relationships and exposure to HIV, particularly among women.

In terms of mental health, the findings are anticipated to show a decline in well-being across all genders, with high rates of anxiety and depressive disorders and posttraumatic stress disorder. Moreover, people living with HIV are expected to be more vulnerable compared to HIV-negative individuals even though they are already facing considerable challenges.

Qualitative findings on health care access may suggest that migrants encounter numerous barriers to obtaining care in French Guiana, including administrative complexity, language and financial barriers, and mobility issues. In response to these challenges and the inadequacies of public services, support from NGOs and community organizations plays a crucial role in assisting migrants with social, health, and legal matters. Health mediators may further facilitate migrants’ access to care, supporting their social integration and empowering them in the process. On the other hand, the proximity between the prevention services delegated by the state to NGOs and newly arrived migrants facilitates early access to screening and prevention. The anthropological results are expected to emphasize the magical-religious perceptions surrounding health and HIV and the role of traditional medicine, including the use of plants in treatment and prevention. Phylogenetic analysis is likely to show a high prevalence of Caribbean B HIV subtypes and community clustering of transmission chains.

For a long time, it was difficult, particularly in France, to carry out surveys on people in relation to their native origin. In this work, after much reflection, the choice was made to work on people born in Haiti. Haiti’s history and very specific geographical situation suggest that Haitians arrive in French Guiana with their own particular baggage (eg, the natural and health disasters of 2010 and 2012). It is always difficult to work on a specific population according to its country of birth without running the risk of adding stigma to the discrimination already experienced by people living with HIV. However, since 2001, several studies have led the scientific community to consider the link between HIV and immigration from the perspective of inequalities [33,34]. A number of indicators point to the even greater difficulties faced by Haitians in French Guiana. Other elements reinforce the idea that Haitian-born people living in French Guiana are subject to particularly precarious situations, inequalities, and even discrimination, all of which are likely to have an impact on their health [35]. Again, keeping in mind the risk of oversimplification, the literature reminds us that health is mediated by social and political processes.

### Limitations

The study methodology has a number of limitations. First, the target population is hidden, most of them undocumented, and the situation (particularly in informal settlements) is rapidly changing. As information on their distribution was incomplete, it was difficult to assess the representativeness of this population in the territory in the absence of up-to-date comparative data. The recruitment sites were selected on a nonprobabilistic and reasoned basis, so selection bias cannot be excluded.

One of the limitations of this study is the strong stigma attached to the Haitian community, particularly the association between the terms “Haiti” and “HIV,” which has a historically negative connotation. This association could reinforce the stigma already attached to this population and limit acceptability.

The recruitment targets for the hepatitis B and HIV groups were ambitious, and the active referral files did not take into account patients lost to follow-up or the growing proportion of patients followed up on in the city (which is difficult to reach).

With regard to the inclusion of participants living with hepatitis B, the follow-up departments for these patients were late in signing up as this study group was added at a later stage, limiting the time available for recruitment.

### Strengths

The biographical approach is the first and principal strength of this work as the data illustrating life courses are embedded in time. This approach makes it possible to study the whole of a person’s life while placing events in the political and social context in which they took place. This approach also limits memory bias by grounding events in key life dates (eg, children’s births). This biographical approach was supplemented by a cross-sectional, qualitative, anthropological approach, and for people living with a chronic virus (HIV or hepatitis B), by a medical questionnaire. For people living specifically with HIV, a phylogenetic analysis was also proposed. All these sources make it possible to triangulate information and reinforce the internal validity of the results.

This is the first in-depth, mixed methodology study on Haitians living in French Guiana, shedding light on a significant population that is key in terms of public health. Despite the fact that the population is hidden and rapidly evolving, French Guiana is a small territory. Collaboration with the main actors in the field, with mediators from the community, and with an anthropologist specialized in French Guiana’s Creole communities increased the chances of obtaining a representative sample of the Haitian population living in French Guiana between 2021 and 2023.

Despite discussions on this point, targeting the Haitian population could be a source of discrimination, particularly when recruiting people living with HIV. In this study, the information and explanations provided by the mediator interviewers helped maximize understanding by building a relationship of trust with the participants, providing them with intelligible information, making them understand what was at stake, and limiting stigmatization. The mediators played a major role in promoting understanding, compliance with ethics, confidentiality, and respect for participants’ rights. The approach taken by community mediators played a major role in the project’s feasibility and the number of included participants. The Parcours d’Haïti mediator community network was undeniably a strength.

### Comparison With Prior Work

The ANRS Parcours 2012 to 2013 survey, a biographical study carried out in the Paris region among migrants from sub-Saharan Africa, was divided into 3 groups equivalent to those presented in this protocol (general population, people living with HBV, and people living with HIV). This study almost achieved its objectives with regard to the group of people living with HIV but fell short with regard to the group of people living with HBV and the general population [36]. This study revealed difficult living conditions on arrival and established the link between these living conditions and the acquisition of HIV in France [36]. The MAKASI study, carried out in 2019 as a follow-up to the ANRS Parcours 2012 to 2013 study among migrants from sub-Saharan Africa far from care, revealed low levels of health coverage and precarious administrative status, including among people who had been in the French territory for several years [37]. Previous surveys carried out in French Guiana show a very high level of food insecurity [38,39]. A number of studies have shown that the health of migrants in French Guiana and hexagonal France is deteriorating [4,40]. In terms of sexual health, recent studies highlight the high level of exposure to sexual violence among migrant women, the high level of transactional relationships used as a survival strategy, and the cumulative violence experienced by this population [4]. In light of recent literature studies published on the American continent, violence (physical and sexual) and the use of transactional sex are identified as one of the main components of the migratory trajectories for women in this area [41-44].

In the ANRS Parcours 2012 to 2013 survey, up to 50% of the people surveyed from sub-Saharan Africa contracted HIV after their arrival in France, and of these, 58% acquired HIV during the settlement period (between 0 and 6 years after migration), and 42% acquired HIV after settling (>6 years after migration) [45]. With regard to HIV, a number of studies have been carried out in French Guiana in recent years (including phylogenetic studies), enabling us to understand the factors driving the epidemic [9-11,14,16,17]. Elements similar to those found in the ANRS Parcours 2012 to 2013 study have been highlighted in these studies, with most foreigners living with HIV having acquired the virus after migrating to French Guiana [10,11]. The studies carried out showed that HIV was diagnosed later in Haitians than in native-born people but was detected more quickly in Haitians than in Surinamese individuals or Brazilians [17]. In light of the recent literature published on the American continent, numerous studies point to inconsistent condom use among migrant populations as well as a lack of knowledge about the risk factors and consequences of STIs [46-48].

### Assumptions and Hypothetical Study Outcomes

#### Quantitative and Biographical Data

In comparison with the results found in the literature, and particularly of the ANRS Parcours 2012 to 2013 study conducted in Paris area, we hypothesize that the study will reveal difficult living conditions after arrival in French Guiana and that these conditions will last for a long time. We will try to confirm that the departures from Haiti were precipitated by historical events and that the routes are more or less complex depending on the path taken. We hypothesize that migratory routes vary according to migration periods. We hypothesize that access to a residence permit is burdensome and that this is a factor negatively impacting the health of people arriving on the Guianese territory. We presume that access to formal housing is very limited and determined by the level of education and knowing someone in French Guiana previously. We hypothesize that relationships are determined by social conditions but that motherhood in women is more determined by age and social representations even when living conditions are precarious. We hypothesize that many people do not have the health coverage to which they are entitled and that health care services, although available, are not necessarily accessible (for reasons that include transport, health coverage, health literacy, and possible discrimination). We will also try to confirm the hypothesis that having contacts with NGOs and social services improves access to care compared to those who have not benefited from this support. We also make the assumptions that people from Haiti experience high levels of food insecurity after arriving in French Guiana and that many people are isolated.

We also hypothesize that the mental health of Haitians living in French Guiana is impaired when living conditions are precarious, violence has occurred, or relatives are separated.

During these years, people may be exposed to situations of sexual vulnerability and to unwanted sexuality and transactional, paid, or even forced sex, exposing them to a greater risk of acquisition of HIV or STIs and psychological trauma. In terms of sexual health, we hypothesize that, regardless of the participant group, a large proportion of the population has been exposed to sexual violence and transactional relationships and that these phenomena particularly concern women. Finally, we hypothesize that the use of contraception is low and that recourse to abortion is very limited.

Concerning the follow-up of people living with HIV and people living with HBV, we hypothesize that Haitian populations in French Guiana use screening relatively early and that those diagnosed with HIV enter care relatively early, in line with the screening and mediation services available in the region, notably through the French Red Cross. We also think that impaired social conditions, social isolation, and low health literacy are associated with a delay in screening. A catch-up certainly exists for women during pregnancy. It is likely that periods of disruption of care are closely linked to the social situation of the people concerned, which changes from year to year. The biographical approach will enable us to confirm the hypothesis that, in the years during which a disruption in care occurred, the person faced social or administrative difficulties more often. In addition, we assume that periods of loss to follow-up are more frequent among people living with HBV, linked to an incomplete understanding of the disease, the absence of therapeutic indications for a large proportion of people living with HBV, prolonged follow-up intervals, and a lower civil society investment than that in people living with HIV. With regard to people living with HIV, we hypothesize that they will report unprotected sex in the years following their infection.

Finally, we hypothesize that conducting a long interview focusing on health issues is likely to contribute to respondents’ access to and use of health care.

#### Qualitative and Anthropological Data

One of the aims of the qualitative study was to gain a better understanding of the cultural representations associated with health within the Haitian community. The relationship between care and life course, especially migration, will also be studied. We hypothesize that this anthropological approach will reveal a number of internal logics specific to Haitian ethnomedicine. It should also lead to a better understanding of the cultural motivations underlying the different ways in which patients seek care and, ultimately, help improve the conditions of prevention and care for Haitian patients.

#### Phylogenetic Analysis

The phylogenetic approach assumes that it will be possible to determine the origin of viruses from published viral sequences (GenBank) and that, from the molecular timeline, it will be possible to reconstruct the history of transmission and the time lag between acquisition and transmission of the virus, which is important information for a better understanding of the particularities of the epidemic in this population. We hypothesize that infections occur most often with sequences compatible with intracommunity transmission before and after arrival and, therefore, that prevention should be carried out at the community level.

### Expected Follow-up

The results of this study are likely to improve our understanding of the sexual vulnerability of people from Haiti with a view to setting up a sexual health pathway incorporating a diversified range of appropriate screening and prevention services. This pathway will help reduce the risk of infection with HIV, HBV, and STIs, as well as unwanted sex and unwanted pregnancies. It will help improve the pathway for entering and remaining in care in general and following the discovery of HIV or chronic HBV infection. It will tie in with work already underway to develop prevention and care services directly in the informal settlements where a large population of Haitians live. The results of this study are likely to help provide medical, psychological, and social support in conjunction with all the institutional and voluntary players.

It will also provide a better understanding of the path taken by people living with HIV or HBV once they have been diagnosed and produce information that can be used to implement measures to prevent them from being lost to follow-up.

The results and resulting proposals will be communicated as a high priority to actors in social support, prevention, and care grouped around the COREVIH Guyane. One action is already underway as part of road map 2 of the French sexual health strategy involving the deployment in French Guiana of nurse-mediator pairs equipped to carry out STI and human papillomavirus point-of-care screening to reach the populations furthest from health structures. The results of the Parcours d’Haïti study will help meet the data needs of this priority population and provide a framework for action.

### Conclusions

The Parcours d’Haïti study is original regarding its methodology (a mixed methods study using biographical methods with community coconstruction), its territory (a French territory in South America), and its study population (a large but vulnerable population). Analyses will test the various hypotheses, notably the potential and significant impact of poor reception conditions on the health of individuals and the persistence of HIV transmission in the region. These expected and original results may lead to an adjustment of the reception policy for Haitian people in French Guiana and to the implementation of efficient sexual health programs. The joint development of these programs with community representatives will be a prerequisite. Ideally, this study could be duplicated in other settings that people from Haiti migrate to (eg, Canada and the United States) and extended to other people on the move facing difficult living conditions on arrival. Finally, the participation of peer investigators (health mediators) for this study improved respect for participants’ rights and data quality. This work is likely to contribute to the development of the profession of health research mediators in the future.

## Appendix

Multimedia Appendix 1 Cross-sectional questionnaire administered electronically using the ODK software. The version included herein was approved by the ethics review board. This appendix also contains the medical questionnaires completed by participants living with HIV or hepatitis B virus.

Multimedia Appendix 2 The biographical grid used to collect retrospective data across the respondent’s entire life course. The version provided here was approved by the ethics review board.

Multimedia Appendix 3 The map used to document the routes taken by participants. The version included in this appendix was approved by the ethics review board.

Multimedia Appendix 4 The questionnaire used to assess participant satisfaction with their experience in the study. The version included in this appendix was approved by the ethics review board.

Multimedia Appendix 5 The interview guides used in the qualitative component of the study, focusing on anthropology and health care–seeking practices. These guides correspond to the version approved by the ethics review board. However, they represent only part of the qualitative data collected; additional insights were obtained through ethnographic observations and other qualitative methods, as detailed in the main article.

## Abbreviations

ANRS: Agence Nationale de Recherches sur le Sida et les hépatites virales (the French National Agency for Research on AIDS and Viral Hepatitis)
Fiocruz: Oswaldo Cruz Foundation
HBV: hepatitis B virus
NGO: nongovernmental organization
STI: sexually transmitted infection

## Data Availability

The questionnaires used are available in the supplementary files. All the other elements used in the writing of this work are available on request.

## References

[1] 286 618 habitants en Guyane au 1er janvier 2021. Institut National des Statistiques et des Etudes Economiques.

[2] (2018). La démographie guyanaise toujours aussi dynamique - Insee Analyses Guyane. Institut National des Statistiques et des Etudes Economiques.

[3] La diversité linguistique marque chaque pan de la culture en Guyane. Institut National des Statistiques et des Etudes Economiques.

[4] Alcouffe L, Huber F, Creton PM, Bitan L, Gonzalez A, Volpellier M, Panfili B, Adenis A, Vignier N (2022). Sexual vulnerability of migrant women in the multicultural context of French Guiana: a societal issue. Front Public Health.

[5] Niveaux de vie et pauvreté en Guyane en 2017: la moitié des guyanais vivent sous le seuil de pauvreté. Institut National des Statistiques et des Etudes Economiques.

[6] En 2020, la natalité reste élevée malgré la crise sanitaire. Institut National des Statistiques et des Etudes Economiques.

[7] (2022). Espérance de vie en. Institut National des Statistiques et des Etudes Economiques.

[8] Les inégalités de santé en Guyane: état des lieux et préconisations. Haut Conseil de la Santé Publique.

[9] Nacher M, Vantilcke V, Parriault MC, Van Melle A, Hanf M, Labadie G, Romeo M, Adriouch L, Carles G, Couppié P (2010). What is driving the HIV epidemic in French Guiana?. Int J STD AIDS.

[10] Nacher M, Adriouch L, Van Melle A, Parriault MC, Adenis A, Couppié P (2018). Country of infection among HIV-infected patients born abroad living in French Guiana. PLoS One.

[11] Arantes I, Bello G, Darcissac E, Lacoste V, Nacher M (2021). Using phylogenetic surveillance and epidemiological data to understand the HIV-1 transmission dynamics in French Guiana. AIDS.

[12] Vignier N, Spira RD, Lert F, Pannetier J, Ravalihasy A, Gosselin A, Lydié N, Bouchaud O, Desgrées du Loû A (2017). [Health care access of Sub-Saharan African migrants living with chronic hepatitis B]. Sante Publique.

[13] Desgrees-du-Lou A, Pannetier J, Ravalihasy A, Le Guen M, Gosselin A, Panjo H, Bajos N, Lydie N, Lert F, Dray-Spira R, PARCOURS Study Group (2016). Is hardship during migration a determinant of HIV infection? Results from the ANRS PARCOURS study of sub-Saharan African migrants in France. AIDS.

[14] Junqueira DM, de Matos Almeida SE (2016). HIV-1 subtype B: traces of a pandemic. Virology.

[15] World Bank open data. The World Bank.

[16] (2019). Bulletin de Santé Publique - Guyane - Surveillance et prévention des infections à VIH et autres infections sexuellement transmissibles. Santé Publique France.

[17] Nacher M, Adenis A, Huber F, Hallet E, Abboud P, Mosnier E, Bideau B, Marty C, Lucarelli A, Morel V, Lacapère F, Epelboin L, Couppié P (2018). Estimation of the duration between HIV seroconversion and HIV diagnosis in different population groups in French Guiana: strategic information to reduce the proportion of undiagnosed infections. PLoS One.

[18] Blaizot R, Armanville F, Michaud C, Boceno C, Dupart O, Pansart C, Niemetzky F, Couppie P, Nacher M, Adenis A, Chosidow O, Duvignaud A (2024). Scabies in French Guiana: quantitative and qualitative factors associated with therapeutic failure. J Eur Acad Dermatol Venereol.

[19] Mahamat A, Louvel D, Vaz T, Demar M, Nacher M, Djossou F (2010). High prevalence of HBsAg during pregnancy in Asian communities at Cayenne Hospital, French Guiana. Am J Trop Med Hyg.

[20] Eubanks A, Parriault MC, Van Melle A, Basurko C, Adriouch L, Cropet C, Nacher M (2018). Factors associated with sexual risk taking behavior by precarious urban migrants in French Guiana. BMC Int Health Hum Rights.

[21] Romelus J, McLaughlin C, Ruggieri D, Morgan S (2024). A narrative review of cervical cancer screening utilization among Haitian immigrant women in the U.S.: health beliefs, perceptions, and societal barriers and facilitators. J Immigr Minor Health.

[22] Guillaume D, Amédée LM, Rolland C, Duroseau B, Alexander K (2023). Exploring engagement in cervical cancer prevention services among Haitian women in Haiti and in the United States: a scoping review. J Psychosoc Oncol.

[23] de Souza JB, Heidemann IT, Walker F, Schleicher ML, Konrad AZ, Campagnoni JP (2021). Vulnerability and health promotion of Haitian immigrants: reflections based on Paulo Freire's dialogic práxis. Rev Esc Enferm USP.

[24] Bastien V, Brenes F (2024). Psychiatric illness in Haitian American immigrants and refugees. J Transcult Nurs.

[25] Marseille BR, Commodore-Mensah Y, Davidson PM, Baker D, D'Aoust R, Baptiste DL (2021). Improving hypertension knowledge, medication adherence, and blood pressure control: a feasibility study. J Clin Nurs.

[26] Vardeman ET, Kennelly EJ, Vandebroek I (2024). Haitian women in New York City use global food plants for women's health. J Ethnobiol Ethnomed.

[27] Hansen M, Andersen TE, Armour C, Elklit A, Palic S, Mackrill T (2010). PTSD-8: a short PTSD inventory. Clin Pract Epidemiol Ment Health.

[28] Kroenke K, Spitzer RL, Williams JB, Löwe B (2009). An ultra-brief screening scale for anxiety and depression: the PHQ-4. Psychosomatics.

[29] Bush K, Kivlahan DR, McDonell MB, Fihn SD, Bradley KA (1998). The AUDIT alcohol consumption questions (AUDIT-C): an effective brief screening test for problem drinking. Ambulatory Care Quality Improvement Project (ACQUIP). Alcohol Use Disorders Identification Test. Arch Intern Med.

[30] Lambert Y, Galindo M, Suárez-Mutis M, Mutricy L, Sanna A, Garancher L, Cairo H, Hiwat H, Bordalo Miller J, Gomes JH, Marchesini P, Adenis A, Nacher M, Vreden S, Douine M (2022). Tailoring mobile data collection for intervention research in a challenging context: development and implementation in the Malakit study. JMIR Form Res.

[31] Allison PD (1982). Discrete-time methods for the analysis of event histories. Sociol Methodol.

[32] Brun-Rambaud G, Alcouffe L, Tareau MA, Adenis A, Vignier N (2023). Access to health care for migrants in French Guiana in 2022: a qualitative study of health care system actors. Front Public Health.

[33] Fassin D (2002). L'invention française de la discrimination. Rev Fr Sci Polit.

[34] Fassin D (2001). L'altérité de l'épidémie. Les politiques du sida à l'épreuve de l'immigration. Rev Eur Migr Int.

[35] Osei L, Vignier N, Nacher M, Laumonnier J, Conan C, Clarke L, Koivogui A, Covis S, Valony L, Basurko C, Wiedner-Papin S, Prual A, Cardoso T, Leneuve-Dorilas M, Alcouffe L, Hcini N, Bernard S, Succo T, Vendittelli F, Elenga N (2024). Small for Gestational age newborns in French Guiana: the importance of health insurance for prevention. Int J Public Health.

[36] Desgrées du Loû A, Lert F, Delfraissy JF, Agence nationale de recherches sur le SIDA (France), Institut de recherche pour le développement (France) (2017). Violences sexuelles: place dans le parcours de vie et relation avec le risque d'infection VIH en France. Parcours : parcours de vie et santé des Africains immigrés en France.

[37] Bousmah MA, Gosselin A, Coulibaly K, Ravalihasy A, Taéron C, Senne J, Gubert F, Desgrées du Loû A, MAKASI Study Group (2023). Immigrants' health empowerment and access to health coverage in France: a stepped wedge randomised controlled trial. Soc Sci Med.

[38] Basurko C, Dupart O, Savy M, Obert-Marby C, Mvogo A, Gonzalez A, Trepont A, Cann L, Boceno C, Osei L, Creton P, Dufit V, Thelusme L, Adenis A, Van-Melle A, Huber F, Nacher M (2023). Hunger in French Guiana's vulnerable urban neighborhoods: a neglected consequence of COVID-19. Food Nutr Bull.

[39] Huber F, Basurko C, Oberlis M, Alcouffe L, Rousseau C, Le Poulain K, Gonzalez A, Osei L, Kpossou K, Vignier N, Boceno C, Wiedner-Papin S (2023). [Hunger in French Guiana, an endemic plague worsened by the health crisis]. Sante Publique.

[40] Prieur C, Dourgnon P, Jusot F, Marsaudon A, Wittwer J, Guillaume S In France, one out of six undocumented immigrants suffers from post-traumatic stress disorder. Quest Déconomie Santé Irdes.

[41] Asakura H (2016). Articulando la violencia y las emociones: las experiencias de las mujeres migrantes centroamericanas residentes en Houston, Texas. Sociológica (Méx).

[42] Estrella Vega MY (2018). Entre la autonomía y la subordinación: significados y perspectivas de la experiencia migratoria de mujeres centroamericanas en tránsito por México. Sociológica (Méx).

[43] Obinna DN (2021). Seeking sanctuary: violence against women in El Salvador, Honduras, and Guatemala. Violence Against Women.

[44] Willers S (2016). Migración y violencia: las experiencias de mujeres migrantes centroamericanas en tránsito por México. Sociológica (Méx).

[45] Gosselin A, Ravalihasy A, Pannetier J, Lert F, Desgrées du Loû A, PARCOURS Study Group (2020). When and why? Timing of post-migration HIV acquisition among sub-Saharan migrants in France. Sex Transm Infect.

[46] Conners EE, Swanson K, Morales-Miranda S, Fernández Casanueva C, Mercer VJ, Brouwer KC (2017). HIV risk behaviors and correlates of inconsistent condom use among substance using migrants at the Mexico/Guatemala border. AIDS Behav.

[47] Kenya S, Carrasquillo O, Fatil M, Jones J, Jean C, Huff I, Kobetz E (2015). Human papilloma virus and cervical cancer education needs among HIV-positive Haitian women in Miami. Womens Health Issues.

[48] Luque JS, Tarasenko YN, Maupin JN, Alfonso ML, Watson LC, Reyes-Garcia C, Ferris DG (2015). Cultural beliefs and understandings of cervical cancer among Mexican immigrant women in Southeast Georgia. J Immigr Minor Health.

