# Supplementary material for: Sexual Health Determinants During the Life Course and Migration of Haitian-Origin People in French Guiana: Protocol for the Parcours d’Haïti Biographical and Transdisciplinary Study
Source: JMIR Res Protoc. 2025 Jun 12;14:e63586. doi: 10.2196/63586 (PMC12203027; doi:10.2196/63586)
Supplement: Multimedia Appendix 1 [file resprot_v14i1e63586_app1.pdf]

ENTRE
OSPITALIER
AYENNE

Précarité et vulnérabilité sexuelle au cours du parcours de vie et de migration des personnes originaires d'Haïti vivant ou non avec le VIH en Guyane française

PARCOURS D'HAÏTI

CODE PROMOTEUR

CAHIER D'OBSERVATION

Version 1.2 du 07/04/2022

Code centre |__|__|__|

Numéro participant |__|__|__|__|

Initiales **(Nom, Prénom)** |__|__|

Groupe **(C, B ou T)** |__|

Promoteur :

Centre hospitalier de Cayenne
Av. des flamboyants –
BP 6006, 97306 CAYENNE CEDEX

Investigateur coordonnateur :

Dr VIGNIER, Nicolas
Centre d'Investigation Clinique Antilles Guyane
Centre hospitalier de Cayenne,
Av. des flamboyants –
BP 6006, 97306 CAYENNE CEDEX
☏: *05 94 39 53 85* nicolas.vignier@ch-cayenne.fr

Centre de Méthodologie et de gestion
Responsable scientifique :

Pr NACHER, Mathieu
CIC Inserm 1424, Centre hospitalier de Cayenne
Centre d'Investigation Clinique Antilles Guyane
Centre hospitalier de Cayenne,
Av. des flamboyants –
BP 6006, 97306 CAYENNE CEDEX
☏: *05 94 39 53 85* cicec@ch-cayenne.fr

Code Participant

|___|___|___| - |___|___|___|___| - |___|___| - |___|
(Code Centre - N° consécutif participant –1ere lettre du prénom et 1ere lettre du nom – C, B ou T)

MODALITES DE REMPLISSAGE DU CAHIER D'OBSERVATION

1. Noter le code à 3 lettres du centre, Noter le numéro consécutif de participant à 4 chiffres, les initiales Prénom Nom et le groupe Cas VIH (C), VHB (B) ou Témoins (T)

2. N'utilisez pas d'abréviation mais écrivez en toutes lettres.

3. Inscrivez un seul caractère par case.

4. Valeurs numériques :
   - cadrez les valeurs numériques à droite
   - n'ajoutez pas de virgules
   - ne laissez pas de cases vides, mettez un zéro.

   Incorrect |_2_|_1_|__| Correct |_0_|_2_|_1_|

5. Lorsque la réponse doit être reportée dans des cases fermées, cochez la case correspondante :

   Par exemple : Oui ☐ Non ☒

6. Dates : indiquez les dates sous la forme Jour-Mois-Année (jj/mm/aaaa). Pour les dates incomplètes : si jour non connu mettre par défaut le chiffre 15 (ex : 15/3/2021) ; si jour et mois inconnus mettre par défaut le 15/06 (ex : 15/06/2021)

7. En cas d'absence de données, cocher la case correspondante :
   ☐NR : non réponse
   ☐NVPR : ne veut pas répondre
   ☐NSP : ne sait pas

8. Chaque erreur doit être barrée d'un trait (la valeur erronée doit rester lisible), corrigée à côté, datée et paraphée (avec les initiales du correcteur) avec un stylo à bille noir. N'utilisez pas de correcteur.

Code Participant
|__|__|__|__|__| - |__|__|__|__| - |__|__| - |__|
*(*Code Centre - N° consécutif participant –1ere lettre du prénom et 1ere lettre du nom – C, B ou T*)*

VERIFICATION DES CRITERES D'ELIGIBILITE

**CRITERES D'INCLUSION**	Oui	Non
Pour les 2 groupes :		
➢ Être né à Haïti, quel que soit la nationalité actuelle	1❑	0❑
➢ Arrivés en Guyane depuis plus de 3 mois	1❑	0❑
➢ Âgé de 18 à 60 ans	1❑	0❑
➢ Ne s'opposant pas à sa participation à l'étude	1❑	0❑
Pour le groupe VIH :		
➢ Vivant avec le VIH et diagnostiqué depuis plus de 3 mois, quel que soit son statut vis-à-vis des hépatites B et C	1❑	0❑
➢ Être suivi par un des médecins partenaires de l'étude des hôpitaux de Cayenne, Kourou ou Saint Laurent du Maroni ou en médecine générale	1❑	0❑
Pour le groupe VHB :		
➢ Vivant avec le VHB et diagnostiqué depuis plus de 3 mois et non co-infecté par le VIH	1❑	0❑
➢ Être suivi par un des médecins partenaires de l'étude des hôpitaux de Cayenne, Kourou ou Saint Laurent du Maroni ou en médecine générale	1❑	0❑
Pour le groupe Témoins :		
➢ Déclarant ne pas être infecté par le VIH ni le VHB	1❑	0❑
➢ Être suivi par un des médecins partenaires de l'étude ou être recruté dans l'espace public d'une des villes ciblées par l'étude (Cayenne, Matoury, Rémire-Montjoly, Macouria, Kourou, Sinnamary, Saint-Laurent-du-Maroni, Mana ou Maripasoula)	1❑	0❑
**CRITERES DE NON-INCLUSION**	Oui	Non
Être incapable de répondre à l'enquêteur en français ou créole haïtien	1❑	0❑
Être sous tutelle ou curatelle ou sous mesure de protection juridique	1❑	0❑
Avoir déjà répondu au questionnaire de l'étude	1❑	0❑

*Critères d'inclusion : toutes les cases « **OUI** » sont cochées*<br>*Critères de non inclusion :toutes les cases « **NON** » sont cochées* ⇨	**Patient éligible**
*Critères d'inclusion : une case « **NON** » est cochée (au minimum)* ⇨<br>*Critères de non inclusion : une case « **OUI** » est cochée (au minimum)*	***Patient Non éligible***

# - INCLUS - QUESTIONNAIRE PARCOURS

Inclus	1❑. Oui<br>2❑. Non
Numéro d'identifiant<br>(Code Centre - N° consécutif participant – 1ere lettre du du prénom et 1ere lettre du nom – C, B ou T pour Cas VIH, VHB ou Témoin)	|___|___|___|___| - |___|___|___| - |___|___| - |___|
Précisez le lieu d'inclusion	………………………………………………..
Groupe d'étude	1❑. VIH<br>2❑. VHB<br>2❑. Témoins
Numéro identifiant de l'enquêteur	|___|___|
Date de l'enquête	|___|___|/ |___|___| / |___|___|___|___|
Langue de passation du questionnaire	1❑. Français<br>2❑. Créole haïtien<br>3❑. Autre, précisez : …………
**INCLUSION ET PROFIL DES PARTICIPANTS**	
L'enquêté est	1❑. Un homme<br>2❑. Une femme<br>3❑. Autre, précisez : …………
Pouvez-vous me dire en quelle année vous êtes né(e) ?	|___|___|___|___| ❑ 99. Ne Sait Pas
Si ne sait pas : Pouvez-vous me dire votre âge ?	|___|___|
Jusqu'à quand êtes-vous allé à l'école ?	1❑. N'est jamais allé à l'école<br>2❑. Maternelle-primaire<br>3❑. Lycée (dont Bac)<br>4❑. Études supérieures<br>98❑. Ne veut pas répondre

	99❑. Ne sait pas
Concernant votre maitrise orale de la langue française, vous êtes :	1❑. Très à l'aise<br>2❑. A l'aise<br>3❑. Moyennement à l'aise<br>4❑. Peu à l'aise<br>5❑. Pas du tout à l'aise<br>98❑. Ne veut pas répondre<br>99❑. Ne sait pas
Savez-vous lire et écrire en français ?	1❑. Oui, tout à fait<br>2❑. Difficilement<br>3❑. Non<br>98❑. Ne veut pas répondre<br>99❑. Ne sait pas

PARTIE I : RECIT BIOGRAPHIQUE, RETROSPECTIVE DE TOUTE LA VIE

Compléter la grille biographique :

GRILLE BIOGRAPHIQUE :

GRILLE : HISTORIQUE RESIDENTIEL -

GRILLE : HISTORIQUE RESIDENTIEL -

ARRIVEE EN FRANCE ET SEJOUR EN FRANCE

A. CONDITIONS D'ARRIVEE EN FRANCE

Nous allons maintenant parler de la période de votre arrivée en Guyane, des conditions dans lesquelles vous avez décidé de partir, et comment vous avez rejoint la Guyane :

ENQ : Considérez ici la première arrivée en France qui compte pour elle/lui comme une arrivée (pas comme un voyage)

En quelle année et quel mois êtes-vous arrivé(e) en Guyane ?	|___|___|___|___| (année). ❑ 99. Ne sait pas<br>au mois de |___|___| (mois). ❑ 99. Ne sait pas<br><br>98❑. Non-réponse
Pour quelle raison, êtes-vous parti d'Haïti ?	1❑. Rejoindre un(e) conjoint(e) ou un(e) fiancé(e)

ENQ : ne pas citer, plusieurs réponses possibles	2❑.Rejoindre vos enfants<br>3❑.Rejoindre un autre membre de la famille (hors conjoint et enfants)<br>4❑.Pour vous marier<br>5❑.Étudier<br>6❑.Chercher un travail<br>7❑.Prendre un emploi déjà identifié<br>8❑.Tenter votre chance / vie meilleure<br>9❑. En raison de l'insécurité en Haïti<br>9❑.Vous étiez menacé(e) dans votre pays<br>10❑. En raison d'une catastrophe naturelle (tremblement, ouragan, etc.)<br>11❑.Pour raisons médicales<br>12❑.Parce que vous êtes venu(e) avec un ou vos parents<br>13❑.Ne sait pas/Non réponse<br>14❑.Pour une autre raison, précisez : ………….<br>98❑. Non réponse
Quelles ont été les conditions de votre migration ?	1❑. Très bonnes<br>2❑. Bonnes<br>3❑. Mauvaises<br>4❑. Très mauvaises<br>98❑. Non réponse
Avec qui êtes-vous arrivé(e) ?<br>*ENQ : ne pas citer, plusieurs réponses possibles*	1❑. Avec conjoint<br>2❑. Avec enfant(s)<br>3❑. Avec parents (père, mère, frères ou sœurs)<br>4❑. Seul(e)<br>5❑. Avec une ou d'autre(s) personne(s)<br>98❑. Non réponse
A ce moment-là, connaissiez-vous déjà des personnes qui vivaient en Guyane ?	1❑.Oui, une ou des personnes de votre famille<br>2❑. Oui, un conjoint ou un fiancé<br>3❑.Oui, un ou des amis ou connaissances<br>4❑. Non<br>98❑. Non réponse
Si vous connaissiez déjà quelqu'un, diriez-vous que…	1❑.Au moins une de ces personnes vous a aidé<br>2❑.Personne ne vous a aidé alors que vous avez demandé de l'aide<br>3❑.On vous a aidé au début, puis vous a demandé des choses en échange

	4❑. On vous a aidé au début, puis on a arrêté de vous aider sans vous demander des choses en échange<br>5❑.Vous n'avez demandé aucune aide.<br>98❑. Non réponse
Quelles sont les personnes sur lesquelles vous pouviez compter à votre arrivée ?<br>*ENQ : ne pas citer, plusieurs réponses possibles*	1❑. Votre conjoint ou petit ami.e<br>2❑. Un ou des personnes de votre famille<br>3❑. Des amis,<br>4❑. Les associations<br>5❑. Des membres de votre église,<br>6❑. Des professionnels de certaines institutions (hôpital, CCAS, AS, etc.)<br>7❑. Autres, précisez : …………<br>8❑. Personne - Aucun soutien – Isolement social
**B. NATIONALITES ET TITRES DE SEJOURS**	
Complétez la grille biographique<br>**GRILLE : NATIONALITES ET TITRES DE SEJOUR -**	
Vous est-il arrivé de faire une demande de séjour pour une raison médicale ?	1❑. Oui<br>0❑. Non<br>98❑. Non réponse<br>99❑. Ne sait pas
Si oui, en quelle année la première fois ?	|___|___|___|___| (année)<br>98❑. Non réponse<br>99❑. Ne sait pas
Si oui, cette année-là ou une autre année, avez-vous obtenu un titre de séjour pour raison médicale ?	1❑. Oui<br>0❑. Non<br>98❑. Non réponse<br>99❑. Ne sait pas
**B- HISTOIRES DES ACTIVITES ET RESSOURCES**	
Complétez la grille biographique<br>**GRILLE : ACTIVITES -**<br>**GRILLE : RESSOURCES.**	
Grille biographique 1 et 2 remplie	1❑. Oui

	0❑. Non<br>98❑. Ne veut pas répondre<br>99❑. Ne sait pas
**SANTE SEXUELLE ET REPRODUCTIVE**	
**A. ENTREE DANS LA SEXUALITE**	
Nous allons maintenant parler de votre santé, de votre histoire et de votre santé sexuelle. Je vais maintenant vous poser des questions plus spécifiques. Certaines questions peuvent ne pas vous concerner et il vous suffira de le dire si c'est le cas. Je vous rappelle que le questionnaire est anonyme, et que si vous ne souhaitez pas répondre c'est possible, cependant si vous décidez de répondre, merci de le faire avec sincérité.	
Quel âge aviez-vous la première fois que vous avez eu un rapport sexuel ?	|___|___| Ans<br>97❑. N'a jamais eu de partenaire<br>98❑. Non réponse<br>99❑. Ne sait pas
Ce premier rapport était quelque chose dont vous aviez envie au moment où cela s'est produit ?<br><br>Ce premier rapport sexuel était quelque chose :<br>*Citer les 3 premiers items, une seule réponse possible*	1❑. Que vous souhaitiez à ce moment là<br>2❑. Que vous avez accepté mais que vous ne souhaitiez pas vraiment<br>3❑. Que vous avez été forcé de faire<br>98❑. Non-réponse<br>99❑. Ne sait plus
Lors de ce premier rapport, Avez-vous utilisé un préservatif ?	1❑. Oui<br>0❑. Non<br>98❑. Non réponse<br>99❑. Ne sait pas
Au cours de votre vie, vous est-il arrivé d'avoir des relations sexuelles avec des hommes ?	1❑. Oui<br>0❑. Non<br>98❑. Non réponse<br>99❑. Ne sait pas
Est-ce qu'il vous arrive d'avoir des relations sexuelles avec des femmes ?	1❑. Oui<br>0❑. Non<br>98❑. Non réponse<br>99❑. Ne sait pas
**C- HISTOIRES DES RELATIONS, GROSSESSES & ENFANTS**	

Nous allons maintenant évoquer les grandes étapes de votre vie affective et familiale: les relations ou unions, les enfants et les grossesses que vous ou votre partenaire avez eus.<br>Cette étude concerne 800 personnes. Les chercheurs ont besoin de prévoir toutes les situations. Certaines questions peuvent ne pas vous concerner et il vous suffira de le dire si c'est le cas.
Parlons maintenant des RELATIONS que vous avez eues et qui ont duré au moins un an, en étant marié(e) ou non, y compris avec des personnes dont vous êtes maintenant séparées ou qui sont décédées
Complétez la grille biographique<br>**GRILLE : RELATIONS LONGUES -**

Est-ce que au moins une de ces relations longues avait d'autres partenaires que vous ?	1❑. Oui<br>0❑. Non<br>98❑. Ne veut pas répondre<br>99❑. Ne sait pas

Parlons maintenant des RELATIONS COURTES que vous avez eues, c'est à dire qui ont duré moins un an ou qui étaient occasionnelles.
Complétez la grille biographique<br>**GRILLE : RELATIONS COURTES -**<br>**GRILLE : RELATIONS CONTRAINTES OU TRANSACTIONNELLES -**

Avez-vous déjà été forcé à avoir des rapports sexuels (viol) ?	1❑. Oui<br>0❑. Non<br>98❑. Ne veut pas répondre<br>99❑. Ne sait pas
Si oui, pouvez-vous nous dire ou cela s'est produit ?<br>*Énoncer les réponses. Plusieurs réponses possibles.*	1❑. Dans le pays d'origine<br>2❑. Pendant le parcours migratoire – Dans un autre pays<br>3❑. Après l'arrivée en Guyane<br>98❑. Ne veut pas répondre
Si oui, ces rapports forcés se sont-ils produits plusieurs fois avec la même personne ?	1❑. Oui<br>0❑. Non<br>98❑. Ne veut pas répondre<br>99❑. Ne sait pas
Avez-vous déjà subi des violences physiques ?	1❑. Oui<br>0❑. Non<br>98❑. Ne veut pas répondre

	99❑. Ne sait pas
Si oui, pouvez-vous nous dire ou cela s'est produit ?<br>*Énoncer les réponses. Plusieurs réponses possibles*	1❑. Dans le pays d'origine<br>2❑. Pendant le parcours migratoire – Dans un autre pays<br>3❑. Après l'arrivée en Guyane<br>98❑. Ne veut pas répondre
Nous allons maintenant parler des enfants et de vos grossesses / pour les hommes : des grossesses de vos compagnes	
Complétez la grille biographique<br>**GRILLE : ENFANTS -**<br>**GRILLE : GROSSESSES –**	
**B. CONTRACEPTION - PRESERVATIFS**<br>**Nous allons maintenant parler des différents moyens d'éviter une grossesse. Nous allons voir ensemble ceux que vous avez pu utiliser.**	
<u>Avant de venir en Guyane</u>, est-ce que vous ou votre (vos) partenaire(s) avez utilisé un moyen pour éviter une grossesse?	1❑. Oui<br>0❑. Non<br>97❑. Non concerné (pas de rapport ou rapports homosexuels)<br>98❑. Non réponse<br>99❑. Ne sait pas
Si oui, quel(s) moyens ou méthode(s) avez-vous utilisé ?<br>*Plusieurs réponses possibles*	❑1❑. Pilule<br>❑2❑. Stérilet<br>❑3❑. Injection (Depo)<br>❑4❑. Implant contraceptif<br>❑5❑. Préservatif masculin<br>❑6❑. Préservatif féminin<br>❑7❑. Ligature des trompes – stérilisation<br>❑8❑. Vasectomie<br>❑9❑. Crèmes spermicides, ovules, éponges, anneaux<br>❑10❑. Pilule du lendemain<br>❑11❑. Le retrait du partenaire avant éjaculation<br>❑12❑. Eviter les rapports sexuels les jours à risque<br>❑13❑. Éviter la pénétration<br>❑14❑. Autre méthode, précisez : ………………….<br>❑ 97❑. Non concerné<br>❑ 98❑. Non réponse<br>❑99❑. Ne sait pas

Depuis votre arrivée en Guyane, est-ce que vous ou votre (vos) partenaire(s) avez utilisé un moyen pour éviter une grossesse ?	1❑. Oui<br>0❑. Non<br>97❑. Non concerné<br>98❑. Non réponse<br>99❑. Ne sait pas
Si oui, quel(s) moyens ou méthode(s) avez-vous utilisé ?<br>*Plusieurs réponses possibles*	❑1. Pilule<br>❑2. Stérilet<br>❑3. Injection (Depo)<br>❑4. Implant contraceptif<br>❑5. Préservatif masculin<br>❑6. Préservatif féminin<br>❑7. Ligature des trompes – stérilisation<br>❑8. Vasectomie<br>❑9. Crèmes spermicides, ovules, éponges, anneaux<br>❑10. pilule du lendemain<br>❑11. le retrait du partenaire avant éjaculation<br>❑12. éviter les rapports sexuels les jours à risque<br>❑13. Éviter la pénétration<br>❑14. Autre méthode, précisez : …………………….<br>❑ 97. Non concerné<br>❑ 98. Non réponse<br>❑99. Ne sait pas
**C. RAPPORTS SEXUELS DES DOUZE DERNIERS MOIS :**<br>**Nous allons maintenant parler de sexualité, et plus précisément des 12 derniers mois**	
Avec combien d'hommes avez-vous eu des rapports sexuels au cours des 12 derniers mois ?	|___|___| partenaires masculins<br>98. Non réponse<br>99. Ne sait pas
Avec combien de femmes avez-vous eu des rapports sexuels au cours des 12 derniers mois ?	|___|___| partenaires féminins<br>98. Non réponse<br>99. Ne sait pas
Si vous avez eu des rapports au cours des douze derniers mois, vous avez utilisé des préservatifs…	1❑. Systématiquement avec tous/ toutes les partenaires<br>2❑. Systématiquement avec certain(e)s mais pas avec les autres<br>3❑. De temps en temps avec tous / toutes les partenaires<br>4❑. De temps en temps avec les un(e)s mais pas avec les autres<br>5❑. Jamais<br>98❑. Non réponse

	99❑. Ne sait pas
Avez-vous déjà eu des ruptures de préservatif ?	1❑. Souvent<br>2❑. Parfois<br>3❑. Rarement<br>4❑. Jamais<br>98❑. Non réponse<br>99❑. Ne sait pas
Avez-vous un désir de grossesse/paternité actuel ?	1❑. Oui<br>0❑. Non<br>98❑. Non réponse<br>99❑. Ne sait pas
Si vous avez eu des rapports avec un homme/une femme (pour les femmes/les hommes) cours des 12 derniers mois, avez-vous utilisé un autre moyen de contraception que le préservatif ?	1❑. Oui<br>0❑. Non<br>97❑. Non concerné<br>98❑. Non réponse<br>99❑. Ne sait pas
Si oui, quel(s) moyens ou méthode(s) avez-vous utilisé au cours des 12 derniers mois ?<br>*Plusieurs réponses possibles*	❑1. Pilule<br>❑2. Stérilet / DIU<br>❑3. Injection de progestatif (Depo®)<br>❑4. Implant contraceptif<br>❑5. Préservatif masculin<br>❑6. Préservatif féminin<br>❑7. Ligature des trompes – stérilisation<br>❑8. Vasectomie<br>❑9. Crèmes spermicides, ovules, éponges, anneaux<br>❑10. Pilule du lendemain<br>❑11. Le retrait du partenaire avant éjaculation<br>❑12. Éviter les rapports sexuels les jours à risque<br>❑13. Éviter la pénétration<br>❑14. Autre méthode<br>❑ 97. Non concerné<br>❑ 98. Non réponse<br>❑99. Ne sait pas
Si oui, êtes-vous satisfait de votre moyen pour éviter une grossesse?	1❑. Très satisfait(e)<br>2❑. Assez satisfait(e)

	3❑. Peu satisfait(e)<br>4❑. Pas satisfait(e) du tout<br>98❑. Non réponse<br>99❑. Ne sait pas
Si vous n'utilisez pas de moyen pour éviter une grossesse en ce moment. Est-ce parce que… ?	1❑. Vous n'avez pas de rapports sexuels en ce moment<br>2❑.Vous souhaitez avoir un enfant<br>3❑.Vous venez d'accoucher ou allaitez<br>4❑.Vous évitez les rapports sexuels les jours à risque où vous utilisez le retrait ou les préservatifs<br>5❑. Vous n'y pensez pas<br>6❑. Vous avez peur des effets secondaires<br>7❑.Aucune méthode contraceptive ne vous convient à vous ou à votre partenaire<br>8❑.Autres situations (ménopause, rapports homosexuels…)<br>9❑. Utilisation systématique du préservatif<br>97❑. Non concerné<br>98❑. Non réponse
Votre/vos partenaire(s) actuels a/ont-ils déjà effectué un test pour le VIH ?	1❑. Oui<br>0❑. Non<br>97❑. N'a pas de partenaire<br>98❑. Non réponse<br>99❑. Ne sait pas
Quel âge a votre partenaire actuel (le plus âgé) ?	|___|___| ans<br>97❑. N'a pas de partenaire<br>98❑. Ne veut pas répondre<br>99❑. Ne sait pas
La santé sexuelle au sens scientifique est définie comme un état de bien-être.<br>Actuellement, vous sentez vous à l'aise dans votre vie sexuelle ?	1❑. Très satisfait(e)/à l'aise<br>2❑. Assez satisfait(e)/à l'aise<br>3❑. Peu satisfait(e)/à l'aise<br>4❑. Pas satisfait(e) du tout/à l'aise<br>98❑. Non réponse
Avez-vous déjà entendu parler d'un traitement d'urgence qui permet de ne pas attraper le VIH après un rapport sans protection (TPE – Traitement Post Exposition) ?	1❑. Oui<br>0❑. Non<br>98❑. Non réponse<br>99❑. Ne sait pas
Savez-vous ce qu'est la PreP – Prophylaxie Pré-exposition au	1❑. Oui

VIH ?	0❑. Non<br>98❑. Non réponse<br>99❑. Ne sait pas
La PrEP est un comprimé préventif qui protège du VIH. Seriez-vous intéressée par la PrEP ?	1❑. Oui<br>0❑. Non<br>98❑. Non réponse<br>99❑. Ne sait pas
Est-ce que vous vous sentez en sécurité chez vous ?	1❑. Oui tout à fait<br>2❑. Oui, globalement<br>3❑. Pas vraiment<br>4❑. Pas du tout<br>98❑. Non réponse
En fonction des réponses à ces questions, les participants pourront se voir remettre une plaquette avec les ressources en santé sexuelle, voir être aider dans la prise d'un rendez-vous rapide si besoin.	
**D- HISTOIRES DES TESTS DE DEPISTAGE**	
Nous allons essayer maintenant de repérer les dépistages que vous avez pu faire au cours de votre vie.<br>Parlons maintenant du dépistage du VIH	
Avez-vous déjà fait un test de dépistage pour le VIH/Sida ?	1❑. Oui<br>0❑. Non<br>98❑. Ne veut pas répondre<br>99❑. Ne sait pas
A quelle occasion a été fait votre premier test de dépistage du VIH après votre arrivée en Guyane ?	1❑. À l'occasion d'un bilan de santé<br>2❑. Parce que le médecin vous l'a proposé<br>3❑. Lors d'une hospitalisation<br>4❑. Lors d'un don de sang<br>5❑. Lors d'un dépistage systématique à la Croix Rouge<br>6❑. Lors d'un dépistage TROD par une association.<br>7❑. Lors d'un bilan de grossesse<br>8❑. Pour avoir vos papiers de séjour<br>9❑. Pour faire un emprunt, obtenir un emploi, voyager dans un autre pays<br>10❑. Parce que vous étiez malade (y compris IST – Infections Sexuellement Transmissibles)<br>11❑. Parce que vous avez voulu savoir

	12❑. Parce que quelqu'un de votre entourage avait le VIH/Sida<br>13❑. Parce que vous avez pris un risque<br>14❑. Parce que vous vouliez arrêter le préservatif<br>16❑. Pourune autre raison.Précisez : …………..<br>98❑. Refus de répondre<br>99❑. Ne sait pas
Complétez la grille biographique<br>**GRILLE - TESTS DE DEPISTAGE - VIH -**	
Avez-vous déjà été dépisté pour l'hépatite B ?	1❑. Oui<br>0❑. Non<br>98❑. Ne veut pas répondre<br>99❑. Ne sait pas
Complétez la grille biographique<br>**GRILLE - TESTS DE DEPISTAGE - HEPATITE B -**	
Pour les femmes :<br>Avez-vous déjà fait un test de dépistagedu cancer du col de l'utérus (frottis cervico-utérin, pap smear, test HPV) ?	1❑. Oui<br>0❑. Non<br>98❑. Ne veut pas répondre<br>99❑. Ne sait pas
Pour les femmes, si oui :<br>A quelle occasion a été fait votre premier test de dépistage du cancer du col de l'utérus après votre arrivée en Guyane ?	1❑. À l'occasion d'un bilan de santé<br>2❑. Parce que le médecin vous l'a proposé<br>3❑. Lors d'une hospitalisation<br>4❑. Lors d'une consultation gynécologique<br>5❑. Lors d'un dépistage systématique à la Croix Rouge<br>6❑. Lors du suivi ou après une grossesse<br>10❑. Parce que vous étiez malade (y compris IST – Infections Sexuellement Transmissibles)<br>11❑. Parce que vous avez voulu savoir<br>12❑. Parce que quelqu'un de votre entourage avait eu un cancer du col de l'utérus<br>15❑. Parce que vous vouliez vous faire vacciner<br>16❑. Pour une autre raison, Préciser : ……………………………………….<br>98❑. Refus de répondre<br>99❑. Ne sait pas@
Complétez la grille biographique	

**GRILLE - TESTS DE DEPISTAGE - HPV -**	
Avez-vous déjà fait des tests de dépistage des autres infections sexuellement transmissibles (Chlamydiae, Gonocoque, Syphilis) ?	1❑. Oui<br>0❑. Non<br>98❑. Ne veut pas répondre<br>99❑. Ne sait pas
Si oui, à quelle année remonte le dernier ?	|___|___|___|___| 0098❑. Ne veut pas répondre<br>0099❑. Ne sait pas
Avez-vous déjà fait eu une infection sexuellement transmissible ?	1❑. Oui<br>0❑. Non<br>98❑. Ne veut pas répondre<br>99❑. Ne sait pas
Si oui, de quelle IST s'agissait-t-il ?<br>*Plusieurs réponses possibles*	❑ 1. VIH/SIDA<br>❑ 2. Hépatite B<br>❑ 3. Hépatite C<br>❑ 4. Syphilis<br>❑ 5. Gonocoque<br>❑ 6. Chlamydia<br>❑ 7. Papillomavirus Humain (HPV)<br>❑ 8. Herpès génital (HSV2)<br>❑ 9. Mycoplasme<br>❑ 10. Autre, précisez : ………………………………..<br>❑ 98. Ne sait pas<br>❑ 99.Pas de réponse
**E- SANTE GENERALE ET HISTOIRE DES MALADIES**<br><br>**Nous allons parler maintenant de votre santé en général.**	
A quel endroit consultez-vous habituellement quand vous en avez besoin ?	1❑. Chez mon médecin traitant<br>2❑. A la PASS<br>3❑. Lors des équipes mobiles sanitaires de MDM<br>4❑. Dans un CPS de la Croix Rouge Française<br>5❑. Auprès d'un médecin de garde<br>6❑. Aux urgences<br>7❑. Chez un tradipraticien / médecin feuille<br>8❑. Autre, précisez :…………………………………..

	98❑. Non réponse<br>99❑. Ne sait pas
Combien de fois avez-vous consulté un médecin généraliste au cours des 12 derniers mois ?<br>*ENQ : tenir compte de toutes les consultations médicales même préventives sauf spécialisées, sage femmes, infirmiers*	|___|___| consultations<br>98❑. Non réponse<br>99❑. Ne sait pas
Pour les femmes :<br>En quelle année avez-vous vu la dernière fois un gynécologue ou une sage femme ?	|___|___|___|___| 0000❑. N'a jamais vu de gynécologue<br>0098❑. Ne veut pas répondre<br>0099❑. Ne sait pas
Avez-vous consulté un ougan/mambo ou un docteur feuille au cours des 12 derniers mois ?	1❑. Oui<br>0❑. Non<br>98❑. Ne veut pas répondre<br>99❑. Ne sait pas
Êtes déjà retourné au pays pour vous soigner ?	1❑. Oui<br>0❑. Non<br>98❑. Ne veut pas répondre<br>99❑. Ne sait pas
En ce moment, comment est votre état de santé en général ?	1❑. Très bon<br>2❑. Bon<br>3❑. Moyen<br>4❑. Mauvais<br>5❑. Très mauvais<br>98❑. Non réponse
Nous allons maintenant parler de votre santé mentale, de comment vous allez psychologiquement.<br>Au cours des 2 dernières semaines, à quelle fréquence avez-vous été gêné(e) par les problèmes suivants ?<br>*ENQ : chaque question peut être posée en deux temps (oui/non et fréquence) si plus clair pour le participant. Une réponse aux quatre questions est nécessaire pour évaluer la santé mentale.*	
Vous vous êtes senti(e) nerveux.se, anxieux.se ou sous tension.	1❑. Jamais<br>2❑. Plusieurs jours (quelques jours)<br>3❑. Plus de la moitié du temps<br>4❑. Presque tous les jours<br>98❑. Non réponse

Vous n'avez pas réussi à contrôler votre inquiétude.	1❑. Jamais<br>2❑. Plusieurs jours (quelques jours)<br>3❑. Plus de la moitié du temps<br>4❑. Presque tous les jours<br>98❑. Non réponse
Vous aviez peu d'intérêt ou de plaisir à faire les choses. Peu de motivation.	1❑. Jamais<br>2❑. Plusieurs jours (quelques jours)<br>3❑. Plus de la moitié du temps<br>4❑. Presque tous les jours<br>98❑. Non réponse
Vous vous êtes senti(e) triste, déprimée, ou désespérée.	1❑. Jamais<br>2❑. Plusieurs jours (quelques jours)<br>3❑. Plus de la moitié du temps<br>4❑. Presque tous les jours<br>98❑. Non réponse
Voici des symptômes que les gens présentent parfois après avoir vécu un événement traumatique, en avoir été témoin ou y avoir été confronté. Pouvez-vous me dire dans quelle mesure certains de ces symptômes vous ont gêné ou vous gênent depuis cet ou ces évènements éventuels :<br>*ENQ : chaque question peut être posée en deux temps (oui/non et fréquence) si plus clair pour le participant. Une réponse aux huits questions est nécessaire pour évaluer la santé mentale*	
Pensées ou souvenirs récurrents de l'évènement.	1❑. Jamais<br>2❑.Rarement<br>3❑.Parfois<br>4❑.La plupart du temps<br>98❑. Non réponse
Sentiment que l'évènement se reproduit.	1❑. Jamais<br>2❑.Rarement<br>3❑.Parfois<br>4❑.La plupart du temps<br>98❑. Non réponse
Cauchemars récurrents à propos de l'évènement.	1❑. Jamais<br>2❑.Rarement<br>3❑.Parfois<br>4❑.La plupart du temps<br>98❑. Non réponse
Réactions émotionnelles ou physiques soudaines lorsqu'on	1❑. Jamais

se rappelle l'événement.	2❑.Rarement<br>3❑.Parfois<br>4❑.La plupart du temps<br>98❑. Non réponse
Évitez les activités qui vous rappellent l'événement.	1❑. Jamais<br>2❑.Rarement<br>3❑.Parfois<br>4❑.La plupart du temps<br>98❑. Non réponse
Éviter les pensées ou les sentiments associés à l'événement.	1❑. Jamais<br>2❑.Rarement<br>3❑.Parfois<br>4❑.La plupart du temps<br>98❑. Non réponse
Sentiment de nervosité, être facilement effrayé.	1❑. Jamais<br>2❑.Rarement<br>3❑.Parfois<br>4❑.La plupart du temps<br>98❑. Non réponse
Je me sens sur mes gardes.	1❑. Jamais<br>2❑.Rarement<br>3❑.Parfois<br>4❑.La plupart du temps<br>98❑. Non réponse
Concernant votre santé de façon plus générale :	
Avez-vous actuellement d'autres maladies chroniques, c'est-à-dire une maladie qui dure longtemps ou qui revient régulièrement?	1❑. Oui<br>0❑. Non<br>98❑. Ne veut pas répondre<br>99❑. Ne sait pas
Si oui, quelle est (sont) cette (ces) maladie(s) chronique(s) ?	………………………………………………………………<br>98❑. Ne veut pas répondre<br>99❑. Ne sait pas
Est-ce que vous avez eu la tuberculose ?	1❑. Oui<br>0❑. Non<br>98❑. Non réponse

	99❑. Ne sait pas
Si oui, est-ce que vous avez pris des comprimés pendant au moins 6 mois pour cette tuberculose ?	1❑. Oui<br>0❑. Non<br>98❑. Non réponse<br>99❑. Ne sait pas
Complétez la grille biographique<br>**GRILLE : MALADIES OU PROBLEMES DE SANTE -**<br>**GRILLE : HOSPITALISATION -**	
**Consommations de substances psychoactives**<br>**Nous allons maintenant aborder votre consommation de tabac, d'alcool et de drogues.**	
Est-ce que vous fumez, ne serait-ce que de temps en temps ?	1❑. Oui, tous les jours<br>2❑. Oui, de temps en temps<br>3❑. Non, je n'ai jamais fumé<br>4❑. Non, j'ai arrêté<br>98❑. Non réponse
Quelle est votre consommation d'alcool (y compris la bière) ? (Fréquence de la consommation).	1❑. Vous ne buvez jamais d'alcool<br>2❑. 1 fois par mois ou moins<br>3❑. 2 à 4 fois par mois<br>4❑. 2 à 3 fois par semaine<br>5❑. Au moins 4 fois par semaine<br>98❑. Non réponse
**Si oui, c**ombien de verres contenant de l'alcool consommez-vous un jour typique où vous buvez ?	1❑. Ne boit jamais d'alcool ou 1 ou 2<br>2❑. 3 ou 4<br>3❑. 5 ou 6<br>4❑. 7, 8 ou 9<br>5❑. 10 ou plus<br>98❑. Non réponse
**Si oui, a**vec quelle fréquence buvez-vous six verres ou davantage lors d'une occasion particulière ?	1❑. Jamais<br>2❑. Moins d'une fois par mois<br>3❑. Une fois par mois<br>4❑. Une fois par semaine<br>5❑. Tous les jours ou presque<br>98❑. Non réponse
Au cours des 3 derniers mois, avez-vous consommé des substances de types cannabis (kali, ganja), cocaïne, crack,	1❑. Oui<br>0❑. Non

opiacés (héroïne), des médicaments psychotropes ou une autre drogue ?	98❑. Non réponse<br>99❑. Ne sait pas
Si oui la(les)quelles ?<br>*ENQ: plusieurs réponses possibles*	❑1 Cannabis (marijuana, joint, herbe, hash, etc.)<br>❑2 Cocaïne (poudre)<br>❑3 Crack (cocaïne base et black joint)<br>❑4 Stimulants de type amphétamine (MDMA, speed, pilules thaï, pilules coupe faim, ectsay, etc.)<br>❑5 Solvants (colle, essence, diluant, etc.)<br>❑6 Calmants ou somnifères (Valium®, Seresta®, Dormicum®, Stilnox®, etc.)<br>❑7 Hallucinogènes (LSD, champignons, PCP, etc.)<br>❑8 Opiacés (héroïne, morphine, méthadone, codéine, buprénorphine, etc.)<br>❑9 Autres substances ou médicaments, précisez : ……………………………………………………………
Et au cours de votre vie, avez-vous déjà consommé du Crack ?	1❑. Oui<br>0❑. Non, jamais<br>98❑. Non réponse<br>99❑. Ne sait pas
Au cours de votre vie, avez-vous pris de la drogue par voie injectable ?	1❑. Oui<br>0❑. Non, jamais<br>98❑. Non réponse<br>99❑. Ne sait pas
**C. PROTECTION MALADIE**<br>**Nous allons maintenant aborder votre couverture maladie, et comment vous étiez assuré au niveau de la santé.**	
Complétez la grille biographique<br>**GRILLE : PROTECTION MALADIE -**	
Et aujourd'hui quel type de protection maladie avez-vous ?	1❑. La CMU complémentaire (CMU/CMU-C/CSS)<br>2❑. L'Aide Médicale d'Etat (AME)<br>3❑. L'Assurance Maladie = Sécurité sociale = Puma<br>4❑. N'a pas de couverture maladie<br>98❑. Non réponse<br>99. Ne sait pas
Avez-vous aujourd'hui une mutuelle santé/ une assurance complémentaire santé ?	1❑. Oui<br>0❑. Non

	98❑. Non réponse<br>99❑. Ne sait pas
Êtes-vous actuellement couvert(e) à 100% pour une affection de longue durée (ALD) ?	1❑. Oui<br>0❑. Non<br>98❑. Non réponse<br>99❑. Ne sait pas
Depuis votre arrivée en Guyane, vous est-il arrivé de ne pas aller consulter un médecin ou un dentiste alors que vous en aviez besoin à ce moment-là ? (Renoncement aux soins)	1❑. Oui<br>0❑. Non<br>98❑. Non réponse<br>99❑. Ne sait pas
Si oui, pour quelles raisons ?<br>*Ne pas citer, plusieurs réponses possibles*	❑1. Raisons financières<br>❑2. Pas de couverture maladie<br>❑3. Difficultés de transport, trop loin<br>❑4. Délai d'attente<br>❑5. Ne sait pas comment faire, où aller<br>❑6. Barrière de la langue<br>❑7. Problèmes de papiers<br>❑8. A peur d'être mal accueillie, discriminée, rejetée<br>❑9. Peur des soins<br>❑10. Vous n'aviez pas le temps d'aller chez le médecin<br>❑11. Autre, précisez : ………………………………….<br>❑98. Non réponse<br>❑99. Ne sait pas
Est-il arrivé qu'on refuse de vous soigner?	1❑. Oui<br>0❑. Non<br>98❑. Non réponse<br>99❑. Ne sait pas
Si oui, selon vous à quoi était lié le fait qu'on vous refuse des soins ?<br>*Ne pas citer, plusieurs réponses possibles*	❑ 1. Votre sexe (le fait d'être un homme ou une femme)<br>❑ 2. Votre état de santé ou un handicap<br>❑ 3. Votre couleur de peau<br>❑ 4. Vos origines ou votre nationalité<br>❑ 5. Votre façon de vous habiller<br>❑ 6. Le lieu où vous vivez, la réputation de votre quartier<br>❑ 7. Parce que vous êtes à la CMU-C/CSS ou à l'AME (pour le soin)<br>❑ 8. Parce que vous êtes séropositif.

	❑ 9. Votre orientation sexuelle<br>❑ 10. Votre usage d'alcool ou de drogue actuel ou passé<br>❑ 11. Vos difficultés à vous exprimer en français<br>❑ 12. En raison de votre infection par le VIH<br>❑ 13. Une autre raison, préciser : …………………..<br>❑ 98. Non réponse
Vous êtes-vous déjà senti moins bien traité en soins du fait de votre origine ?	1❑. Oui, souvent<br>2❑. Oui, parfois<br>3❑. Plutôt pas<br>4❑. Non, jamais<br>98❑. Non réponse<br>99❑. Ne sait pas
Avez-vous déjà été accompagné par les structures suivantes :	❑ 1. Médecin du monde<br>❑ 2. Croix Rouge Française<br>❑ 3. PASS<br>❑ 4. Le Comede<br>❑ 5. CCAS<br>❑ 6. PMI<br>❑ 7. Entraides<br>❑ 8. DAAC<br>❑ 9. Conseil départemental d'accès aux droits<br>❑ 10. Association d'aide aux victimes (AGAV)<br>❑ 11. Non, aucune de ces structures
**ACCOMPAGNEMENT SOCIAL, BIEN-ETRE ET EVENEMENTS MARQUANTS**	
Complétez la grille biographique<br>**GRILLE –ACCOMPAGNEMENT SOCIAL ET STRUCTURES FREQUENTEES -**<br>**GRILLE - BIEN-ETRE -**	
Concernant vos réserves alimentaires, au cours des 30 derniers jours, est ce qu'il est arrivé qu'il n'y ait rien du tout à manger dans votre ménage ?<br>*Énoncer les réponses*	0❑. Jamais<br>1❑. Rarement (1 à 2 fois)<br>2❑. Parfois (3 à 10 fois)<br>3❑. Souvent (plus de 10 fois)<br>98❑. Pas de réponse<br>99❑. Ne sait pas

Au cours des 30 derniers jours, est ce qu'il vous est arrivé de vous coucher en ayant faim, vous ou un membre de votre foyer (parce que vous n'aviez pas assez mangé dans la journée) ?<br>*Énoncer les réponses*	0□. Jamais<br>1□. Rarement (1 à 2 fois)<br>2□. Parfois (3 à 10 fois)<br>3□. Souvent (plus de 10 fois)<br>98❑. Pas de réponse<br>99❑. Ne sait pas
Au cours des 30 derniers jours, est ce qu'il vous est arrivé de passer toute une journée et une nuit sans manger (vous ou l'un des membres de votre ménage) ? (Énoncer les réponses)	0❑. Jamais<br>1❑. Rarement (1 à 2 fois)<br>2❑. Parfois (3 à 10 fois)<br>3❑. Souvent (plus de 10 fois)<br>98❑. Pas de réponse<br>99❑. Ne sait pas
Complétez la grille biographique<br>**GRILLE - FAIM -**<br>**GRILLE - EVENEMENTS MARQUANTS -**	
**PARTIE IV : RESEAU SOCIAL ACTUEL**<br>**Concernant votre vie actuelle, et votre entourage :**	
Aujourd'hui vous habitez avec :<br>*Plusieurs réponses possibles*	❑1.Votre partenaire<br>❑2. Votre(vos) enfant(s)<br>❑3. Vos parents<br>❑4. Vos frères et soeurs<br>❑5. Vos neveux et nièces<br>❑6. Vos tantes et oncles<br>❑7. Vos grands parents<br>❑8. D'autres compatriotes<br>❑9. D'autres personnes<br>❑ 10. Seul.e<br>❑98. Non réponse<br>❑99. Ne sait pas
En cas de difficultés, y-a-t-il dans votre entourage des personnes sur qui vous puissiez compter pour vous héberger quelques jours en cas de besoin ?	1❑. Oui<br>0❑. Non<br>98. Non réponse<br>99. Ne sait pas

En cas de difficultés, y-a-t-il dans votre entourage des personnes sur qui vous puissiez compter pour vous apporter une aide matérielle (y compris un prêt d'argent) ?	1❑. Oui<br>0❑. Non<br>98❑. Non réponse<br>99❑. Ne sait pas

**MODULE PVVIH**<br>*Si groupe = 1, poursuivre sur le module VIH*	
Si vous avez déjà eu une période de rupture de plus de 6 mois, pour quelle(s) raison(s) avez-vous arrêtez votre suivi ?<br>*Ne pas citer, plusieurs réponses possibles*	❑ 1.Je ne croyais pas que j'étais malade<br>❑ 2.Vous n'aviez pas de papier ou votre droit au séjour était périmé<br>❑ 3.Vous n'aviez pas ou plus de couverture maladie<br>❑ 4.Votre situation financière était compliquée<br>❑ 5.Vous n'aviez plus de logement<br>❑ 6.Vous étiez parti à l'étranger<br>❑ 7.Vous étiez incarcéré<br>❑ 8.C'était une période de forte consommation de drogues<br>❑ 9.Vous n'alliez pas bien (souffrance psychologique)<br>❑ 10. L'hôpital était trop loin ou trop difficile d'accès<br>❑ 11. Vous étiez inquiet de ce que les gens pensent, ou que les gens vous voient ou s'aperçoivent de votre maladie<br>❑ 12. Vous aviez d'autres préoccupations quotidiennes comme le travail, l'école, les questions administratives ou l'aide aux enfants ou aux personnes passent avant la prise en charge de votre maladie<br>❑ 13. Le médecin ou l'équipe de soins ne vous avais pas dit qu'il fallait venir<br>❑ 14. Vous avez oublié votre rendez vous<br>❑ 15. Vous n'avez pas réussi à prendre rendez-vous<br>❑ 16. Vous ne pensiez pas qu'un suivi était nécessaire car on ne vous a pas expliqué<br>❑ 17. Vous ne vouliez pas revenir car vous avez vécu une mauvaise expérience relationnelle avec l'équipe de l'hôpital<br>❑ 18. Vous étiez suivi ailleurs (précisez où et quand) dans la colonne remarque de la grille biographique<br>❑ 19. J'avais recours à la médecine traditionnelle<br>❑ 20. Autre, précisez : …

	❑ 97. Non concerné (pas de période de rupture de suivi)<br>❑98. Non réponse<br>❑99. Ne sait pas
Si concerné : Pour quelle(s) raison(s) avez-vous repris votre suivi ensuite ?<br>*Ne pas citer, plusieurs réponses possibles*	❑ 1.Complication médicale<br>❑ 2.Ma famille ou mes proches m'ont encouragé à reprendre mon suivi<br>❑ 3.Consultation pour un autre motif<br>❑ 4.Prise de conscience de l'importance du suivi<br>❑ 5.Retour de l'étranger<br>❑ 6.On m'a recontacté par téléphone<br>❑ 7.On m'a recontacté par courrier<br>❑ 8.Mon médecin traitant m'a recontacté<br>❑ 9.Sortie de prison ou de rétention<br>❑ 10. Autre, précisez : …<br>❑ 97. Non concerné (pas de période de rupture de suivi)<br>❑98. Non réponse<br>❑99. Ne sait pas
Est-ce que votre suivi a été impacté par la période du Covid ?	1❑. Oui, beaucoup<br>2❑. Oui, un peu<br>3❑. Plutôt pas<br>4❑. Non, pas du tout<br>98❑. Non réponse
Quelle est la durée du trajet entre votre domicile et l'hôpital ou votre lieu de suivi habituel ?	1❑.< 1 heure<br>2❑.1 à 3 heures<br>3❑.3 à 12 heures<br>4❑.> 12 heures<br>98❑. Ne sait pas<br>99❑.Pas de réponse
Quel moyen de transport utilisez-vous pour venir en consultation à l'hôpital ou à votre lieu de suivi habituel ?<br>*Ne pas citer, plusieurs réponses possibles*	❑ 1. Marche<br>❑2. Vélo<br>❑3. Scooter<br>❑4. Voiture personnelle<br>❑5. Taxico<br>❑6. Bus public<br>❑7. Un.e ami.e m'aide<br>❑98. Non réponse

	❑99. Ne sait pas
Vous estimez que l'obtention d'un rendez-vous de suivi de votre maladie est quelque chose de :	0❑.Très facile<br>1❑.Facile<br>2❑.Moyennement facile<br>3❑.Difficile<br>4❑. Très difficile<br>❑98. Non réponse<br>❑99. Ne sait pas
Si la prise de rendez-vous est difficile, pouvez m'expliquez pourquoi ?	……………………………………………………………
A combien évaluez-vous votre niveau de confiance avec le traitement que vous prenez ?	0❑.Nulle<br>1❑.Faible<br>2❑.Moyenne<br>3❑.Forte<br>4❑.Très forte<br>❑98. Non réponse<br>❑99. Ne sait pas
Avez-vous recours à une médecine traditionnelle / à un médecin feuille dans la prise en charge de votre infection ?	1❑. Oui<br>0❑. Non<br>98❑. Ne veut pas répondre<br>99❑. Ne sait pas
Pensez-vous que certaines plantes sont des traitements efficaces contre le VIH ?	1❑. Oui<br>0❑. Non<br>98❑. Ne veut pas répondre<br>99❑. Ne sait pas
Et en quelle année pensez-vous avoir été contaminé/infecté par le VIH ?	|___|___|___|___| ❑ 98. Non réponse<br>99❑. Ne sait pas
Pourquoi pensez-vous avoir été infecté(e) à ce moment-là ?	……………………………………………………………<br>……………………………………………………………<br>98❑. Ne veut pas répondre<br>99❑. Ne sait pas
Est-ce que quelqu'un de votre entourage a été informé que vous aviez le VIH ?	1❑. Oui par vous-même<br>2❑. Oui par quelqu'un avec votre accord<br>3❑. Oui par quelqu'un sans votre accord

	4❑. Non<br>98❑. Non réponse<br>99❑. Ne sait pas
Est-ce que votre partenaire actuel est séropositif(ve) ?	1❑. Oui<br>2❑. Non<br>3❑. N'a pas de partenaire<br>98❑. Non réponse<br>99❑. Ne sait pas
Est-ce que votre/vos partenaire actuel sait que vous êtes séropositif(ve) ?	1❑. Oui<br>2❑. Non<br>3❑. N'a pas de partenaire<br>98❑. Non réponse<br>99❑. Ne sait pas
Si votre partenaire actuel est informé que vous avez le VIH, comment a-t-il réagi quand il/elle a appris votre séropositivité ?	1❑. Il/elle a été compréhensif et vous a aidé(e)<br>2❑. Il/elle est devenu(e) plus distant(e)<br>3❑. Il/elle s'est mis(e) en colère et a crié<br>4❑. Il/elle a été violent(e) et vous a frappé(e)<br>5❑. Non réponse<br>6❑. Autre
Est-ce que, globalement, votre entourage a changé d'attitude à votre égard depuis qu'il est informé de votre séropositivité?	1❑. Oui, sont plus proches de vous<br>2❑. Oui, sont plus distants<br>3❑. Non, rien n'a changé<br>4❑. Je ne sais pas<br>5❑. Autre<br>98❑. Non réponse
Est-ce qu'un médecin a déjà refusé de vous soigner car vous vivez avec le VIH ?	1❑. Oui<br>0❑. Non<br>98❑. Ne veut pas répondre<br>99❑. Ne sait pas
Avez-vous bénéficié de l'accompagnement d'une infirmière d'éducation thérapeutique de l'hôpital ?	1❑. Oui<br>0❑. Non<br>98❑. Ne veut pas répondre<br>99❑. Ne sait pas
Avez-vous bénéficié de l'accompagnement d'une infirmière à domicile ?	1❑. Oui<br>0❑. Non<br>98❑. Ne veut pas répondre

	99❑. Ne sait pas
Avez-vous déjà participé à des groupes de parole de personnes vivant avec le VIH ?	1❑. Oui<br>0❑. Non<br>98❑. Ne veut pas répondre<br>99❑. Ne sait pas

MODULE PVVHB<br>Si groupe = 2, poursuivre sur le module VHB	
Si vous avez déjà eu une période de rupture de plus de 6 mois, pour quelle(s) raison(s) avez-vous arrêtez votre suivi ?<br>*Ne pas citer, plusieurs réponses possibles*	❑ 1.Je ne croyais pas que j'étais malade<br>❑ 2.Vous n'aviez pas de papier ou votre droit au séjour était périmé<br>❑ 3.Vous n'aviez pas ou plus de couverture maladie<br>❑ 4.Votre situation financière était compliquée<br>❑ 5.Vous n'aviez plus de logement<br>❑ 6.Vous étiez parti à l'étranger<br>❑ 7.Vous étiez incarcéré<br>❑ 8.C'était une période de forte consommation de drogues<br>❑ 9.Vous n'alliez pas bien (souffrance psychologique)<br>❑ 10. L'hôpital était trop loin ou trop difficile d'accès<br>❑ 11. Vous étiez inquiet de ce que les gens pensent, ou que les gens vous voient ou s'aperçoivent de votre maladie<br>❑ 12. Vous aviez d'autres préoccupations quotidiennes comme le travail, l'école, les questions administratives ou l'aide aux enfants ou aux personnes passent avant la prise en charge de votre maladie<br>❑ 13. Le médecin ou l'équipe de soins ne vous avais pas dit qu'il fallait venir<br>❑ 14. Vous avez oublié votre rendez vous<br>❑ 15. Vous n'avez pas réussi à prendre rendez-vous<br>❑ 16. Vous ne pensiez pas qu'un suivi était nécessaire car on ne vous a pas expliqué<br>❑ 17. Vous ne vouliez pas revenir car vous avez vécu une mauvaise expérience relationnelle avec l'équipe de l'hôpital<br>❑ 18. Vous étiez suivi ailleurs (précisez où et quand) dans la colonne remarque de la grille biographique<br>❑ 19. J'avais recours à la médecine traditionnelle

	❑ 20. Autre, précisez : …<br>❑ 97. Non concerné (pas de période de rupture de suivi)<br>❑98. Non réponse<br>❑99. Ne sait pas
Si concerné : Pour quelle(s) raison(s) avez-vous repris votre suivi ensuite ?<br>*Ne pas citer, plusieurs réponses possibles*	❑ 1.Complication médicale<br>❑ 2.Ma famille ou mes proches m'ont encouragé à reprendre mon suivi<br>❑ 3.Consultation pour un autre motif<br>❑ 4.Prise de conscience de l'importance du suivi<br>❑ 5.Retour de l'étranger<br>❑ 6.On m'a recontacté par téléphone<br>❑ 7.On m'a recontacté par courrier<br>❑ 8.Mon médecin traitant m'a recontacté<br>❑ 9.Sortie de prison ou de rétention<br>❑ 10. Autre, précisez : …<br>❑ 97. Non concerné (pas de période de rupture de suivi)<br>❑98. Non réponse<br>❑99. Ne sait pas
Est-ce que votre suivi a été impacté par la période du Covid ?	1❑. Oui, beaucoup<br>2❑. Oui, un peu<br>3❑. Plutôt pas<br>4❑. Non, pas du tout<br>98❑. Non réponse
Quelle est la durée du trajet entre votre domicile et l'hôpital ou votre lieu de suivi habituel ?	1❑.< 1 heure<br>2❑.1 à 3 heures<br>3❑.3 à 12 heures<br>4❑.> 12 heures<br>98❑. Ne sait pas<br>99❑.Pas de réponse
Quel moyen de transport utilisez-vous pour venir en consultation à l'hôpital ou à votre lieu de suivi habituel ?<br>*Ne pas citer, plusieurs réponses possibles*	❑ 1. Marche<br>❑2. Vélo<br>❑3. Scooter<br>❑4. Voiture personnelle<br>❑5. Taxico<br>❑6. Bus public<br>❑7. Un.e ami.e m'aide

	❑98. Non réponse<br>❑99. Ne sait pas
Vous estimez que l'obtention d'un rendez-vous de suivi de votre maladie est quelque chose de :	0❑.Très facile<br>1❑.Facile<br>2❑.Moyennement facile<br>3❑.Difficile<br>4❑. Très difficile<br>❑98. Non réponse<br>❑99. Ne sait pas
Si la prise de rendez-vous est difficile, pouvez m'expliquez pourquoi ?	…………………………………………………………..
Prenez-vous ou avez-vous pris un traitement médicamenteux pour votre infection par le virus de l'hépatite B ?	1❑. Oui, actuellement<br>2❑. Oui, avant mais plus maintenant<br>0❑. Non, jamais<br>98❑. Ne veut pas répondre<br>99❑. Ne sait pas
Si prend un traitement<br>A combien évaluez-vous votre niveau de confiance avec le traitement que vous prenez ?	0❑.Nulle<br>1❑.Faible<br>2❑.Moyenne<br>3❑.Forte<br>4❑.Très forte<br>❑98. Non réponse<br>❑99. Ne sait pas
Avez-vous recours à une médecine traditionnelle / à un médecin feuille dans la prise en charge de votre infection ?	1❑. Oui<br>0❑. Non<br>98❑. Ne veut pas répondre<br>99❑. Ne sait pas
Pensez-vous que certaines plantes sont des traitements efficaces contre le VHB ?	1❑. Oui<br>0❑. Non<br>98❑. Ne veut pas répondre<br>99❑. Ne sait pas
Et en quelle année pensez-vous avoir été contaminé/infecté par le VHB ?	|___|___|___|___| ❑ 98. Non réponse<br>99❑. Ne sait pas
Pourquoi pensez-vous avoir été	……………………………………………………………

infecté(e) à ce moment-là ?	……………………………………………………………………<br>98❑. Ne veut pas répondre<br>99❑. Ne sait pas
Est-ce que quelqu'un de votre entourage a été informé que vous étiez porteur du VHB ?	1❑. Oui par vous-même<br>2❑. Oui par quelqu'un avec votre accord<br>3❑. Oui par quelqu'un sans votre accord<br>4❑. Non<br>98❑. Non réponse<br>99❑. Ne sait pas
Est-ce que votre partenaire actuel est porteur du VHB ?	1❑. Oui<br>2❑. Non<br>3❑. N'a pas de partenaire<br>98❑. Non réponse<br>99❑. Ne sait pas
Est-ce que votre/vos partenaire actuel sait que vous êtes porteur du VHB ?	1❑. Oui<br>2❑. Non<br>3❑. N'a pas de partenaire<br>98❑. Non réponse<br>99❑. Ne sait pas
Si votre partenaire actuel est informé que vous êtes porteur du VHB, comment a-t-il réagi quand il/elle a appris votre statut ?	1❑. Il/elle a été compréhensif et vous a aidé(e)<br>2❑. Il/elle est devenu(e) plus distant(e)<br>3❑. Il/elle s'est mis(e) en colère et a crié<br>4❑. Il/elle a été violent(e) et vous a frappé(e)<br>5❑. Non réponse<br>6❑. Autre
Est-ce que, globalement, votre entourage a changé d'attitude à votre égard depuis qu'il est informé de votre statut ?	1❑. Oui, sont plus proches de vous<br>2❑. Oui, sont plus distants<br>3❑. Non, rien n'a changé<br>4❑. Je ne sais pas<br>5❑. Autre<br>98❑. Non réponse
Est-ce qu'un médecin a déjà refusé de vous soigner car vous vivez avec le VHB ?	1❑. Oui<br>0❑. Non<br>98❑. Ne veut pas répondre<br>99❑. Ne sait pas
Avez-vous bénéficié de	1❑. Oui

l'accompagnement d'une infirmière d'éducation thérapeutique de l'hôpital ?	0❑. Non<br>98❑. Ne veut pas répondre<br>99❑. Ne sait pas
Avez-vous bénéficié de l'accompagnement d'une infirmière à domicile ?	1❑. Oui<br>0❑. Non<br>98❑. Ne veut pas répondre<br>99❑. Ne sait pas
Avez-vous déjà participé à des groupes de parole de personnes vivant avec le VHB ?	1❑. Oui<br>0❑. Non<br>98❑. Ne veut pas répondre<br>99❑. Ne sait pas

Remerciements<br><br>Remise du kit hygiene et de la plaquette ressources<br><br>Aide à l'orientation si besoin

ÉTUDE « PARCOURS D'HAITI » - QUESTIONNAIRE MEDICAL - PATIENT SUIVI POUR UNE INFECTION VIH

DATE : /__/__/ /__/__/ /__/__/__/__/ ☐**NON RENSEIGNE** ☐**NON FAIT**

Nom du médecin : ……………………………….

Poids : (kg) **/___/___/___/** **Taille** (cm) : **/___/___/___/**

Depuis quand ce(cette) patient(e) est-il(elle) suivi(e) dans ce service ?

☐C'est la première fois ☐Depuis moins d'un an
☐Depuis 1 à 5 an(s) ☐Depuis plus de 5 ans
☐Non renseigné

Son suivi est :

☐Régulier ☐Irrégulier, sans longues périodes de perte de vue ☐Irrégulier, avec longues périodes de perte de vue ☐Non renseigné

Date de diagnostic VIH-1 : Mois : /___/___/ Année : /___/___/___/___/

Date du 1er génotype de résistance réalisé : Jour : /___/___/Mois : /___/___/ Année : /___/___/___/___/

Si non réalisé à Pasteur, précisez le laboratoire de réalisation : ……………….. ☐Non réalisé

Sous type viral :☐ B ☐Non B ➔ préciser le sous type en clair :………………………………………☐Non renseigné

CD4 lors du premier bilan connu

Date : .... , ☐Non renseigné

CD4 (en nombre absolu/mm^3) : ....,☐Non renseigné

Antécédents de pathologie classant Sida

☐Oui☐Non ☐Non renseigné
↳ **Si oui, année du premier événement classant Sida :** /___/___/___/___/.......... Non renseigné ☐

Traitement antirétroviral

☐Traitement ARV en cours☐Traitement interrompu☐ Patient naïf ☐Non renseigné

Nombre de lignées thérapeutiques : /___/___/ ☐Non renseigné

Année du 1er traitement ARV/___/___/___/___/ Non renseigné ☐

Nadir des CD4

Date : .... , ☐Non renseigné

CD4 (en nombre absolu/mm^3) : ....,☐Non renseigné

Charge virale VIH et CD4 lors du dernier bilan connu

Date : ……………………., ☐Non renseigné

Charge virale : ☐ Indétectable → Seuil de détection : ....
☐ Détectable
→ Valeur (en copies/ml) : ....
→ Valeur (en log) : ....
☐ Non renseigné

Date : .... ,☐Non renseigné

CD4 (en nombre absolu/mm^3) : .... Non renseigné ☐

CD4 (en %) : .... Non renseigné ☐

A déjà eu une sérologie syphilis positive ?

☐Oui☐Non ☐Non renseigné

Si oui, année de la première positive : /___/___/___/___/☐Non renseigné

Le test non tréponémique (VDRL ou équivalent) était-t-il également positif : ☐Oui☐Non ,☐ NR

A déjà eu une PCR Chlamydiae positive ?

☐Oui☐Non ☐Non renseigné

Si oui, année de la première positive : /___/___/___/___/ ,☐Non renseigné

A déjà eu une PCR gonocoque positive ?

☐Oui☐Non ☐Non renseigné

Si oui, année de la première positive : /___/___/___/___/,☐Non renseigné

Test de dépistage de l'hépatite B ?

☐ Oui ☐ Non ☐Non renseigné

↳**Statut vis-à-vis de l'hépatite B ?** :

☐ Porteur chronique de l'hépatite B (antigène HBs positif)
↳Année de diagnostic :/___/___/___/___/ Non renseigné ☐

☐ Hépatite B guérie spontanément ou après traitement
(anticorps anti-HBs et anti-HBc positifs, antigène HBs négatif ou indéterminé)

☐ Statut indéterminé (anticorp anti-HBc positif isolé)

☐ Vacciné contre l'hépatite B et pas d'antécédent d'hépatite B
(anticorps anti-HBs positifs, anticorps anti-HBc négatifs)

☐ Pas d'antécédent d'hépatite B ni de vaccination contre l'hépatite B
(tous les antigènes et anticorps de la sérologie sont négatifs)

Antécédent d'hépatite C ?

☐ Oui ☐ Non Non renseigné ☐

↳Année de diagnostic :/___/___/___/___/ Non renseigné ☐

↳Statut actuel ? ☐Hépatite C chronique

☐ Hépatite C guérie spontanément ou après traitement

☐ Non renseigné

↳Traitement : ☐Non renseigné dans le dossier

☐Aucun traitement

☐Traitement en cours ou terminé depuis moins de 6 mois

☐Traitement terminé depuis plus de 6 mois

↳ **Résultat du traitement :**☐succès ☐ échec

☐Non renseigné

Antécédents d’histoplasmose?

☐Histoplasmose active ou en cours de traitement
☐Antécédent personnel d’histoplasmose guérie
☐Aucun antécédent personnel d’histoplasmose
☐Non renseigné

Antécédents de tuberculose ?

☐Tuberculose active ou en cours de traitement
☐Antécédent personnel de tuberculoseguérie
☐Aucun antécédent personnel de tuberculose
☐Non renseigné

Le patient est-t-il atteint de une ou plusieurs des affections suivantes ?

☐Diabète de type 1 ou 2
☐Hypertension artérielle
☐Maladie cardiovasculaire avérée
☐Dépression
☐Autres troubles psychologiques ou psychiatriques
☐Autres, précisez : ....
☐Aucun
☐Non renseigné

Code Participant

|___|___|___|___| - |___|___|___| - |___|___| - |___|
(Code Centre - N° consécutif participant –1ere lettre du prénom et 1ere lettre du nom – C, B ou T)

ÉTUDE « PARCOURS D'HAITI » - QUESTIONNAIRE MEDICAL - PATIENT SUIVI POUR UNE INFECTION CHRONIQUE PAR LE VHB

DATE : /__/__/ /__/__/ /__/__/__/__/ ☐**NON RENSEIGNE** ☐**NON FAIT**

Nom du médecin : ……………………………….

Poids : (kg) **/___/___/___/** **Taille** (cm) : **/___/___/___/**

<u>Depuis quand ce(cette) patient(e) est-il(elle) suivi(e) dans ce service ?</u>

☐C'est la première fois ☐Depuis moins d'un an
☐Depuis 1 à 5 an(s) ☐Depuis plus de 5 ans
☐Non renseigné

<u>Son suivi est :</u>

☐Régulier☐Irrégulier, sans longues périodes de perte de vue ☐Irrégulier, avec longues périodes de perte de vue☐Non renseigné

<u>Date de diagnostic Antigène HBs+ :</u> Mois : /___/___/ Année : /___/___/___/___/

<u>1er bilan connu</u> Date : ....

Transaminases ALAT (en ***x la Normale, 1 si normales)*** : /___/___/___/./___/ Non renseigné ☐
ADN VHB en ui/ml /___/___/___/___/___/___/___/___/ Non renseigné ☐
AcHBe: ☐ positif, ☐ négatif, ☐ Non renseigné AgHBe : ☐ positif, ☐ négatif, ☐ Non renseigné

<u>Évaluation de la fibrose</u>

Ponction Biopsie Hépatique : ☐ Oui ☐ Non
Fibroscan : ☐ Oui ☐ Non
Fibrotest/Fibrometre/Fib-4 ou équivalent: ☐ Oui ☐ Non
<u>Si Oui</u>, dernier résultat :
Score métavir Actité /___/ / Fibrose : /___/ ou élastométrie /___/___/./___/ kPa - Année : /___/___/___/___/

Cirrhose (en cours ou ATCD) : ☐ Oui ☐ Non ☐Non renseigné
Cancer hépatique (en cours ou ATCD) : ☐ Oui ☐ Non ☐ Non renseigné

<u>Traitement</u> : ☐ Aucun traitement initié ☐ Traitement interrompu
☐ Traitement en cours ⇨ **type de traitement : ………………………………………………........**
☐ **Non renseigné**

<u>Si traitement : année du 1er traitement antiviral</u> /___/___/___/___/

<u>Résultats les plus récents</u> Date : . /__/__/ /__/__/ /__/__/__/__/
Transaminases ALAT ***(xN)*** : /___/___/___/./___/ Non renseigné☐
ADN VHB *(en ui/ml)* : /___/___/___/___/___/___/___/___/ Non renseigné ☐

Antécédents de sérologie VIH ?
☐ Non, jamais ☐ Oui, dans les 12 derniers mois ☐ Oui, mais il y a plus de 12 mois ☐ Non renseigné

Si oui, résultat de la dernière sérologie ?
☐ VIH+ ☐ VIH– ☐ Non renseigné

Antécédent d'hépatite C ?
☐ Oui ☐ Non Non renseigné ☐

↳Année de diagnostic :/___/___/___/___/ Non renseigné ☐

↳Statut actuel ? ☐Hépatite C chronique

☐ Hépatite C guérie spontanément ou après traitement

☐ Non renseigné

↳Traitement : ☐Non renseigné dans le dossier

☐Aucun traitement

☐Traitement en cours ou terminé depuis moins de 6 mois

☐Traitement terminé depuis plus de 6 mois

↳ **Résultat du traitement :**☐succès ☐ échec

☐Non renseigné

A déjà eu une sérologie syphilis positive ?

☐Oui☐Non ☐Non renseigné

Si oui, année de la première positive : /___/___/___/___/☐Non renseigné

Le test non tréponémique (VDRL ou équivalent) était-t-il également positif : ☐Oui☐Non ,☐ NR

A déjà eu une PCR Chlamydiae positive ?

☐Oui☐Non ☐Non renseigné

Si oui, année de la première positive : /___/___/___/___/ ,☐Non renseigné

A déjà eu une PCR gonocoque positive ?

☐Oui☐Non ☐Non renseigné

Si oui, année de la première positive : /___/___/___/___/,☐Non renseigné

Antécédents d'histoplasmose ?

☐Histoplasmose active ou en cours de traitement
☐Antécédent personnel d'histoplasmose guérie
☐Aucun antécédent personnel d'histoplasmose
☐Non renseigné

Antécédents de tuberculose ?

☐Tuberculose active ou en cours de traitement
☐Antécédent personnel de tuberculoseguérie
☐Aucun antécédent personnel de tuberculose
☐Non renseigné

Le patient est-t-il atteint d'une ou plusieurs des affections suivantes ?

☐Diabète de type 1 ou 2
☐Hypertension artérielle
☐Maladie cardiovasculaire avérée
☐Dépression
☐Autres troubles psychologiques ou psychiatriques
☐Autres, précisez : ....
☐Aucune
☐Non renseigné
