# Supplementary material for: Sexual Health Determinants During the Life Course and Migration of Haitian-Origin People in French Guiana: Protocol for the Parcours d’Haïti Biographical and Transdisciplinary Study
Source: JMIR Res Protoc. 2025 Jun 12;14:e63586. doi: 10.2196/63586 (PMC12203027; doi:10.2196/63586)
Supplement: Multimedia Appendix 2 [file resprot_v14i1e63586_app2.pdf]

CENTRE HOSPITALIER CAYENNE
Andrée Rosemon

Précarité et vulnérabilité sexuelle au cours du parcours de vie et de migration des personnes originaires d'Haïti vivant ou non avec le VIH en Guyane française

PARCOURS D'HAÏTI

V1.3 du 06/12/2022

GRILLE BIOGRAPHIQUE

NOM DE L'ENQUETEUR : …………………………………………….

N° DE L'ENQUETEUR : |___|___|

DATE DE L'ENTRETIEN : |___|___| |___|___| |___|___|___|___|

N° D'ANONYMAT : |___|___|___|___| |___|___|___| |___|___| |___|

|___|___|___|___| |___|___|___| |___|___| |___|

ANNEES	AGES	1- HISTORIQUE RESIDENTIEL					1.7 NATIONALITES & TITRES DE SEJOUR
			Logement en France				
				1.3 Nature de l'habitat (R2.1)	1.4 Eau courante (R2.2)	1.6 Comment avez-vous payé le loyer? (R2.4)	
		1.1 PAYS (R0)	1.2 COMMUNE DE RESIDENCE EN GUYANE (R1) 1.2 Précisez si zone d'habitat informel: ZAINF	1- propre logement<br>2- hébergé par famille<br>3- hébergé par d'autre.s personne.s<br>4- hébergé structure collective (précisez)<br>5- Squat/Rue<br>6-Instabilité résidentielle<br>7- autre, préciser	Oui<br>Non	ARGT: avec mon argent<br>SERV: hébergé contre service<br>SEX: hébergé contre rapports sexuel<br>AUTRE: autre, précisez	1.7 Titres de séjour (Tit1.1-4)<br>Aucun<br>Visa<br>DA<br>Récépissé<br>TS<1an<br>TS 1-3 ans<br>TS 10 ans<br>Nationalité FR<br>OQTF<br>Autre (préciser)
2023							
2022							
2021							
2020							
2019							
2018							
2017							
2016							
2015							
2014							
2013							
2012							
2011							
2010							
2009							
2008							
2007							
2006							
2005							
2004							
2003							
2002							
2001							
2000							
1999							
1998							
1997							
1996							
1995							
1994							
1993							
1992							
1991							
1990							
1989							
1988							
1987							
1986							
1985							
1984							
1983							
1982							
1981							
1980							
1979							
1978							
1977							
1976							
1975							
1974							
1973							
1972							
1971							
1970							
1969							
1968							
1967							
1966							
1965							
1964							
1963							

|___|___|___|___| |___|___|___| |___|___| |___|

ANNEES	2. HISTORIQUE DES ACTIVITES			
		SI AU TRAVAIL		
	**2.1** **Que faisiez-vous...? Quelle était votre principale occupation? (A1)**		**Activités en France**	
	1- Inactif<br>2- Etudiant / Ecole<br>3- Jobs (non déclarés, dont agricole pour autrui)<br>4- Activité salarié<br>5- Activité indépendante<br>7- Agriculture/pêche/abatti (pour soi)<br>8- Retraité<br>9- Autre (précisez)	**2.2** Métier exercé (en clair) (A2)	**2.3** **Si salarié, qu'aviez vous comme type de contrat? (A3)**<br>1. Un CDI<br>2. Un CDD, Interim<br>3. Pas de contrat	**2.4** **D'où venaient vos ressources financières? (Re1)** *(plusieurs codes possibles pour une même période)*<br>1. de votre propre activité<br>2. de l'activité de votre conjoint/partenanire<br>3. de la solidarité de la famille<br>4. de la solidarité communautaire<br>5. d'allocations de l'état (préciser: RSA, CAF, Chomage, ADA, AAH, Invalidite, Bourses, Autres)<br>6. Aucunes ressources
2023				
2022				
2021				
2020				
2019				
2018				
2017				
2016				
2015				
2014				
2013				
2012				
2011				
2010				
2009				
2008				
2007				
2006				
2005				
2004				
2003				
2002				
2001				
2000				
1999				
1998				
1997				
1996				
1995				
1994				
1993				
1992				
1991				
1990				
1989				
1988				
1987				
1986				
1985				
1984				
1983				
1982				
1981				
1980				
1979				
1978				
1977				
1976				
1975				
1974				
1973				
1972				
1971				
1970				
1969				
1968				
1967				
1966				
1965				
1964				
1963				

|___|___|___|___| |___|___|___| |___|___| |___|

ANNEES	3.<br>HISTOIRE DES RELATIONS			4.<br>GROSSESSES et ENFANTS			
	**3.1**<br>**Relations longues (plus d'1 an) (Rel1)**<br><br>Rel1 (Relation 1)<br>Rel2 (Relation 2)<br>, etc.<br>CO: cohabitant<br>ETR: vit à l'étranger<br>MAR: Marié<br>Si aucune, écrire "AUCUNE" en tête de colonne	**3.2**<br>**Relations courtes ou occasionnelles (Rco1)**<br><br>RCO (Relation courte)<br>Si aucune, écrire "AUCUNE" en tête de colonne	**3.3**<br>**Relations contraintes ou payantes**<br><br>TRANSAC: transactionnelle<br>PAYES: payés<br>FORCES: Rapport forcés<br>CONJUG: violence conugale<br>PAYANTS: recours prostitution<br>Si aucune, écrire "AUCUNE" en tête de colonne	**4.1**<br>**Enfants & Grossesses (Enf1.1, Enf1.6, Gr1.1-1.5)**<br>E1 (1er enfant)<br>E2 (2ème enfant), etc.<br>DCE1 (si décès 1er enfant)<br>G1 (1ere grossesse)<br>G2 (2eme grossesse), etc.<br>G -IVG (avortement provoqué)<br>G -FCS (Fausse couche spontanée)<br>G -MN (Mort né)<br>Si aucun, écrire "AUCUN" en tête de colonne	**4.2**<br>**Souhait d'enfant & grossesse (Enf1.2, Gr1.3)**<br><br>Oui<br>Non<br>NR / NSP	**4.3**<br>**Au moment où la grossesse a débuté, faisiez vous qq chose pour éviter une grossesse? (Enf1.3, Gr1.4)**<br>Oui, Non	**4.4**<br>**Où vit cet enfant actuellement? (Enf1.4)**<br>1. En France avec vous<br>2. En France mais pas avec vous<br>3. Au pays<br>4. Dans un autre pays<br>5. L'enfant est décédé<br>6. Autre<br>98. Non réponse<br>99. Ne sait pas
2023							
2022							
2021							
2020							
2019							
2018							
2017							
2016							
2015							
2014							
2013							
2012							
2011							
2010							
2009							
2008							
2007							
2006							
2005							
2004							
2003							
2002							
2001							
2000							
1999							
1998							
1997							
1996							
1995							
1994							
1993							
1992							
1991							
1990							
1989							
1988							
1987							
1986							
1985							
1984							
1983							
1982							
1981							
1980							
1979							
1978							
1977							
1976							
1975							
1974							
1973							
1972							
1971							
1970							
1969							
1968							
1967							
1966							
1965							
1964							
1963							

|___|___|___|___| |___|___|___| |___|___| |___|

ANNEES	5. TESTS DE DEPISTAGE			6. SUIVI VIH	7. HISTOIRE DES MALADIES ET HOSPITALISATION	8. PROTECTION MALADIE
	**5.1**<br>**VIH (Vih1.1-2)**<br>VIH1neg (1er test negatif)<br>VIH2pos (2eme test positif)<br>VIH1? (1er test résultat inconnu)<br>Si aucun, écrire "AUCUN" en tête de colonne	**5.2**<br>VHB (Vhb1.1-2)<br>VHB1neg (1er test negatif)<br>VHB2pos (2eme test positif)<br>VHB1? (1er test résultat inconnu)<br>Si aucun, écrire "AUCUN" en tête de colonne	**5.3**<br>**Col utérus/HPV (Hpv1.1-5)**<br>HPV1neg (1er frottis negatif)<br>HPV2pos (2ème frottis anormal)<br>HPV1? (1er test résultat inconnu)<br>SUIVI-HPV Si aucun, écrire "AUCUN" en tête de colonne	**6.1**<br>**SuiviVIH-VHB (Svih1.1-3)**<br>INF-VIH / INF-VHB (infection probable)<br>DIAG-VIH / DIAG-VHB (diagnostic VIH)<br>SUIVI-VIH / SUIVI-VHB (début de suivi)<br>TTT-VIH / TTT-VHB (date début traitement)<br>Si aucun suivi, écrire "AUCUN" en tête de colonne	**7.1**<br>Diabète (Exemple de maladie)<br>HOP (hospitalisation)<br>Si aucune, écrire "AUCUNE" en tête de colonne	**8**<br>**Couvertures maladies (Cm1.1-4)**<br>CMU-C (CMU/CMU-C/CSS)<br>AME (Aide Médicale d'Etat)<br>AssMal (Assurance Maladie = sécurité sociale = Puma)<br>COUVMA (couverture maladie de nature inconnue)<br>AUCUNE (Pas de couverture)
2023						
2022						
2021						
2020						
2019						
2018						
2017						
2016						
2015						
2014						
2013						
2012						
2011						
2010						
2009						
2008						
2007						
2006						
2005						
2004						
2003						
2002						
2001						
2000						
1999						
1998						
1997						
1996						
1995						
1994						
1993						
1992						
1991						
1990						
1989						
1988						
1987						
1986						
1985						
1984						
1983						
1982						
1981						
1980						
1979						
1978						
1977						
1976						
1975						
1974						
1973						
1972						
1971						
1970						
1969						
1968						
1967						
1966						
1965						
1964						
1963						

|___|___|___|___| |___|___|___| |___|___| |___|

ANNEES	9. Accompagnement social	10. Bien-être	11. Faim	12. Evenements maquants
	**9**<br>**Accompagnement social et structures fréquentées (As1.1-6)**<br>AS (Assistant social)<br>Asso (Association): précisez<br>Media (Médiateur)<br>CCAS (Centre communal d'action social)<br>AUTRE, précisez en clair<br>Si aucun, écrire "AUCUN" en tête de colonne	**10**<br>**Bien-être (Be1.1)**<br>"D" années difficiles<br>"TD" années très difficiles<br>NSP: ne sait plus	**11**<br>**Faim (Fm1.1)**<br>FAIM (être aller se coucher le soir en ayant faim)	**12**<br>**Evenements marquants (Em1.1-)**<br>EVTM (Evenement marquant)<br>CRA (centre de rétention administratif)<br>INCAR (Incarcération))<br>OQTF (obligation quitter territoire)<br>AUTRE: autre, précisez<br>Si aucun, écrie "AUCUN" en tête de colonne
2023				
2022				
2021				
2020				
2019				
2018				
2017				
2016				
2015				
2014				
2013				
2012				
2011				
2010				
2009				
2008				
2007				
2006				
2005				
2004				
2003				
2002				
2001				
2000				
1999				
1998				
1997				
1996				
1995				
1994				
1993				
1992				
1991				
1990				
1989				
1988				
1987				
1986				
1985				
1984				
1983				
1982				
1981				
1980				
1979				
1978				
1977				
1976				
1975				
1974				
1973				
1972				
1971				
1970				
1969				
1968				
1967				
1966				
1965				
1964				
1963				

|___|___|___|___| |___|___|___| |___|___| |___|

ANNEES	13. REMARQUES ET PRECISIONS
2023	
2022	
2021	
2020	
2019	
2018	
2017	
2016	
2015	
2014	
2013	
2012	
2011	
2010	
2009	
2008	
2007	
2006	
2005	
2004	
2003	
2002	
2001	
2000	
1999	
1998	
1997	
1996	
1995	
1994	
1993	
1992	
1991	
1990	
1989	
1988	
1987	
1986	
1985	
1984	
1983	
1982	
1981	
1980	
1979	
1978	
1977	
1976	
1975	
1974	
1973	
1972	
1971	
1970	
1969	
1968	
1967	
1966	
1965	
1964	
1963	
