# Supplementary material for: Sexual Health Determinants During the Life Course and Migration of Haitian-Origin People in French Guiana: Protocol for the Parcours d’Haïti Biographical and Transdisciplinary Study
Source: JMIR Res Protoc. 2025 Jun 12;14:e63586. doi: 10.2196/63586 (PMC12203027; doi:10.2196/63586)
Supplement: Multimedia Appendix 4 [file resprot_v14i1e63586_app4.pdf]

CENTRE HOSPITALIER CAYENNE
Andrée Rosemon

Précarité et vulnérabilité sexuelle au cours du parcours de vie et de migration des personnes originaires d'Haïti vivant ou non avec le VIH en Guyane française

PARCOURS D'HAÏTI

CODE PROMOTEUR

CAHIER D'OBSERVATION – Volet Satisfaction / Parcours de soins

Version 1.3 du 06/12/2022

**Code centre**	└─┴─┴─┘
**Numéro participant**	└─┴─┴─┘
**Initiales(Nom, Prénom)**	└─┴─┘
**Groupe (C, B ou T)**	└─┘

Promoteur :

Centre hospitalier de Cayenne
Av. des flamboyants –
BP 6006, 97306 CAYENNE CEDEX

Investigateur coordonnateur :

Dr VIGNIER, Nicolas
Centre d'Investigation Clinique Antilles Guyane
Centre hospitalier de Cayenne,
Av. des flamboyants –
BP 6006, 97306 CAYENNE CEDEX
☏: *05 94 39 53 85* dr.vignier@gmail.com

Centre de Méthodologie et de gestion
Responsable scientifique :

Pr NACHER, Mathieu
CIC Inserm 1424, Centre hospitalier de Cayenne
Centre d'Investigation Clinique Antilles Guyane
Centre hospitalier de Cayenne,
Av. des flamboyants –
BP 6006, 97306 CAYENNE CEDEX
☏: *05 94 39 53 85* mathieu.nacher@ch-cayenne.fr

Code Participant

|___|___|___| - |___|___|___| - |___|___| - |___|

(Code Centre - N° consécutif participant –1ere lettre du prénom et 1ere lettre du nom – C, B ou T)

MODALITES DE REMPLISSAGE DU CAHIER D'OBSERVATION

1. Noter le code à 3 lettres du centre, Noter le numéro consécutif de participant à 3 chiffres, les initiales Prénom Nom et le groupe Cas VIH (C), VHB (B) ou Témoins (T)

2. N'utilisez pas d'abréviation mais écrivez en toutes lettres.

3. Inscrivez un seul caractère par case.

4. Valeurs numériques :
   - cadrez les valeurs numériques à droite
   - n'ajoutez pas de virgules
   - ne laissez pas de cases vides, mettez un zéro.

   Incorrect |_2_|_1_|__| Correct |_0_|_2_|_1_|

5. Lorsque la réponse doit être reportée dans des cases fermées, cochez la case correspondante :

   Par exemple : Oui ☐ Non ☒

6. Dates : indiquez les dates sous la forme Jour-Mois-Année (jj/mm/aaaa). Pour les dates incomplètes : si jour non connu mettre par défaut le chiffre 15 (ex : 15/3/2021) ; si jour et mois inconnus mettre par défaut le 15/06 (ex : 15/06/2021)

7. En cas d'absence de données, cocher la case correspondante :
   - ☐NR : non réponse
   - ☐NVPR : ne veut pas répondre
   - ☐NSP : ne sait pas

8. Chaque erreur doit être barrée d'un trait (la valeur erronée doit rester lisible), corrigée à côté, datée et paraphée (avec les initiales du correcteur) avec un stylo à bille noir. N'utilisez pas de correcteur.

Code Participant
|___|___|___| - |___|___|___| - |___|___| - |___|
(Code Centre - N° consécutif participant –1ere lettre du prénom et 1ere lettre du nom – C, B ou T)

PARCOURS DE SOINS ET SATISFACTION PARTICIPATION PARCOURS

**SATISFACTION**		
Date de l'appel téléphonique : |___|___| / |___|___| / |___|___||___|___| date_appel		
Appelant : [en clair]<br>appelant		
Personne injoignable au :<br>(plusieurs réponses possibles)<br>☐ Premier rappel<br>☐ Deuxième rappel<br>☐ Troisième rappel<br>☐ Numéro non attribué ou non disponible<br>injoignablex		
Il y a quelques mois vous avez participé à l'enquête PARCOURS, nous vous recontactons afin de voir avec vous, ce que vous avez pu retirer de cette participation.		
Vous avez participé à l'enquête PARCOURS, vous en souvenez-vous ?<br>part_parcours	Oui ❑<br>(1)	Non ❑<br>(2)
Concernant l'entretien que vous avez eu avec la médiatrice-enquêtrice, vous diriez que vous êtes :<br>satisf		
☐ Très satisfait (4)<br>☐ Satisfait (3)<br>☐ Moyennement satisfait (2)<br>☐ Peu satisfait (1)<br>☐ Pas satisfait (0)		
*Si Moyennement, Peu ou Pas satisfait : Pouvez me préciser pourquoi ? [en clair]<br>passatisf		
**ORIENTATION**		
Lors de cet entretien, avez-vous bénéficié d'une orientation ? Vous as-t-ont proposé d'aller vers un service, une structure particulière ou un soignant ?<br>orientation	Oui ❑<br>(1)	Non ❑<br>(2)
Si oui, la ou lesquelles ? (plusieurs réponses possibles)<br>structure_orientationx<br>❑ (1). Médecin du monde<br>❑ (2). Croix Rouge Française<br>❑ (3). PASS<br>❑ (4). Le Comede		

❑ (5). CCAS<br>❑ (6). PMI<br>❑ (7). Entraides<br>❑ (8). DAAC<br>❑ (9). Conseil départemental d'accès aux droits<br>❑ (10). Association d'aide aux victimes (AGAV)<br>❑ (11). CGSS<br>❑ (12). Médecin généraliste<br>❑ (13). Autre, Précisez : …………………….. structure_autre		
Si oui, vous êtes-vous rendu auprès de cette ou ces structures ? rendu_orientation<br>❑ (1). Oui<br>❑ (2). Pour une partie des orientations seulement, Précisez :……………………………………………………… ………………………………… rendu_autre<br>❑ (3). J'en ai eu l'intention mais j'ai renoncé. Précisez pourquoi :…………………………………………………… …………………………………. rendu_renoncement<br>❑ (4). Non		
Si oui, la ou les orientations ont-t-elles été bénéfiques pour vous ? benef_orientation	Oui ❑ (1)	Non ❑ (0)
Si vous avez bénéficié d'une orientation, avez-vous été bien reçu dans la structure vers laquelle vous avez été orienté ? acceuil	Oui ❑ (1)	Non ❑ (0)
Si vous n'aviez pas de couverture maladie au moment de l'enquête, avez-vous pu obtenir une couverture maladie depuis l'entretien ? couvmalpost	Oui ❑ (1)	Non ❑ (0) Non concerné ❑ (99)
Si aviez renoncé à des soins avant l'entretien, avez-vous pu voir un médecin depuis l'entretien ? consmedpost	Oui ❑ (1)	Non ❑ (0) Non concerné ❑ (99)
Pour les femmes : Avez-vous réalisé un (nouveau) dépistage du cancer du col de l'utérus / frottis depuis l'entretien ? depistagepost	Oui ❑ (1)	Non ❑ (0)
Avez-vous réalisé un (nouveau) dépistage du VIH et des IST depuis l'entretien ? depistagepost	Oui ❑ (1)	Non ❑ (0)
Avez-vous reçu des informations sur la PrEP au cours de l'entretien ?	Oui ❑ (1)	Non ❑ (0)

Code Participant
|___|___|___|___| - |___|___|___|___| - |___|___|___| - |___|
(Code Centre - N° consécutif participant –1ere lettre du prénom et 1ere lettre du nom – C, B ou T)

informationpreptpe		
Étiez-vous intéressé par la PrEP à l'issu de l'entretien ? interet_prep	Oui ❑ (1)	Non ❑ (0)
Si oui, avez-vous pu bénéficier de la prescription de la PrEP ? prescription_prep	Oui ❑ (1)	Non ❑ (0)
Si non, pour quelle raison : raison_nonprep<br>…………………………………………………………..…..…..…..…..…..		
**PARTICIPATION A L'ENQUETE**		
Diriez-vous que la participation à PARCOURS a été bénéfique pour vous ? benef_part		
☐ Oui, très bénéfique (3)<br>☐ Oui, assez (2)<br>☐ Pas plus que ça (1)<br>☐ Non, m'a fait du mal (0)<br>☐ Pas de réponse (99)		
Pourquoi ? (Choix multiple, plusieurs réponses possibles) motif_benefx<br>☐ Je me suis sentie écouté (16)<br>☐ J'ai pu être conseillé (15)<br>☐ J'ai parlé de choses desquelles je n'avais jamais parlé (14)<br>☐ Je me suis senti en confiance (13)<br>☐ J'ai pu avoir accès à des informations (12)<br>☐ J'ai pu avoir accès a ce dont j'avais besoin (11)<br>☐ J'ai pu avoir des préservatifs (10)<br>☐ J'ai eu un kit d'hygiène (9)<br>☐ J'ai beaucoup ris (8)<br>☐ Je ne me suis pas senti en confiance (7)<br>☐ Je me suis sentie mal à l'aise (6)<br>☐ Je n'ai pas compris pourquoi on me posait toutes ces questions (5)<br>☐ Je n'ai pas souhaité parler des sujets proposés (4)<br>☐ J'ai eu peur que les informations sur moi circulent (3)<br>☐ J'ai perdu du temps (3)<br>☐ Je ne me suis pas senti respecté (2)<br>☐ Les conditions n'étaient pas bonnes (1)<br>☐ Ça ne m'a rien apporté (0)<br>☐ Autre, précisez : (98)<br>……………………………………………..…..…..…..…..…..<br>motif_autre		
Accepteriez-vous de participer à une autre étude du même type ?	Oui ❑ (1)	Non ❑ (2)

-
