# Supplementary material for: Sexual Health Determinants During the Life Course and Migration of Haitian-Origin People in French Guiana: Protocol for the Parcours d’Haïti Biographical and Transdisciplinary Study
Source: JMIR Res Protoc. 2025 Jun 12;14:e63586. doi: 10.2196/63586 (PMC12203027; doi:10.2196/63586)
Supplement: Multimedia Appendix 5 [file resprot_v14i1e63586_app5.pdf]

Pratiques et représentations médicales des personnes originaires d'Haïti vivant ou non avec le VIH ou une infection chronique par le VHB en Guyane française. Étude qualitative ancillaire du projet « PARCOURS D'HAÏTI » Guide d'entretien Professionnels

Numéro d'entretien : …………..
Date de l'entretien : …………….
Lieu de l'entretien : …………….
Numéro identifiant : |_P_| - |___|___|___| - |___|___|

1 - Données personnelles :

1.1 Âge :

1.2 Sexe :

1.2 Lieu de naissance (et date d'arrivée en Guyane le cas échéant) :

1.3 Profession et missions :

1.4 Ancienneté d'exercice :

1.5 Lieu ou structure d'exercice

2 Recours aux soins

2.1 Que pouvez me dire au sujet des personnes migrantes en Guyane ?

2.2 Qu'en est-t-il de l'accès aux soins après l'arrivée en Guyane ?

2.3 Et en termes d'accès à la prévention ?

2.4 Quelles sont les barrières spécifiques à l'accès aux soins des personnes migrantes ?

2.5 Pensez-vous que les personnes migrantes ont souvent recours à la médecine traditionnelle ou à la phyothérapie ?

2.6 Que pensez-vous de ces pratiques ?

2.7 A votre avis, qu'elle représentation ont les personnes migrantes de la biomédecine / médecine moderne ?

2.8 Avez-vous la notion de discriminations dans les structures de santé ou de prévention en Guyane ?

2.9 Quelle est le niveau d'insécurité alimentaire des personnes que vous accompagnez et comment les publics s'adaptent ?

3 Représentations autour du VIH et du VHB

Nous allons maintenant parler du l'hépatite B.
3.1 Que pouvez-vous me dire concernant l'hépatite B ?

Si besoin, apport d'information sur la pathologie et sa prévention.
3.2 Quel est le niveau de connaissance des populations migrantes concernant l'hépatite B et sa prévention ?

3.3 Qu'en est-t-il de la vaccination contre l'hépatite B ?

3.4 Accompagnez-vous des personnes vivant avec une infection chronique par l'hépatite B ?

3.5 Si oui, quels sont les enjeux de leur parcours de soins ?

Nous allons maintenant parler du VIH
3.6 Que pouvez-vous me dire concernant le VIH ?

Si besoin, apport d'information sur la pathologie et sa prévention.
3.7 Quel est le niveau de connaissance des populations migrantes concernant l'infection par le VIH et sa prévention ?

3.8 Qu'en est-t-il de la prévention du VIH par la PrEP ?

3.9 Accompagnez-vous des personnes vivant avec le VIH ?

3.10 Si oui, quels sont les enjeux de leur parcours de soins ?

3.11 Selon vous, quel est la meilleure façon de prévenir ces maladies en Guyane ?

3.12 Souhaitez vous ajouter quelque chose ?

Pratiques et représentations médicales des personnes originaires d'Haïti vivant ou non avec le VIH ou une infection chronique par le VHB en Guyane française.
Étude qualitative ancillaire du projet « PARCOURS D'HAÏTI »
Guide d'entretien Usagers

Numéro d'entretien : ....
Date de l'entretien : ....
Lieu de l'entretien : ....
Numéro identifiant : |_U_| - |___|___|___| - |___|___|

1 - Données personnelles :

1.1 Âge :

1.2 Sexe :

1.2 Lieu de naissance (et date d'arrivée en Guyane le cas échéant) :

1.3 Niveau scolaire :

Primaire	
Secondaire	
Etud sup	
Non scolarisé	

1.9 Situation familiale :

Célibataire	
En couple	
Célibataire	
En couple	

1.10 Parentalité

Parent	N :
Sans enfant	

1.10 Activité actuelle :

Etudiant	Salarié	Indépendant	Jobs	Inactif

1.11 Maladie chronique (y compris infection par le VIH et le VHB)

Oui, précisez :	
Non	

2 Recours aux soins

2.1 Parlez-moi de votre arrivée en Guyane ? Comment s'est passée votre arrivée sur le territoire ?

2.2 Depuis votre arrivée, avez-vous fait quelque chose pour rester en bonne santé, pour la prévention ?

2.3 En cas de maladie, allez-vous d'abord essayer de voir un médecin, ou d'abord prendre un remède? Pourquoi ?

2.4 Avez-vous déjà rencontré des difficultés à accéder aux soins ? Pourquoi ?

2.5 Avez-vous déjà eu l'occasion de vous soigner à la fois avec des remèdes et avec des médicaments pharmaceutiques ? Décrire l'itinéraire thérapeutique suivi par le patient :

2.6 Comment s'est passé ce traitement multiple (le cas échéant) ? Y a-t-il eu des interactions, des mélanges entre médecine moderne et phytothérapie ?

2.7 Comment percevez-vous la médecine occidentale, hospitalière ? Les soignants ?

2.8 Avez-vous éprouvé des difficultés (accueil, incompréhension, remarques…) dans vos expériences relationnelles avec les soignants ?

2.9 Pensez-vous que l'alimentation soit un facteur de bonne santé important ? Pourquoi ?

2.10 Eprouvez-vous des difficultés à vous nourrir correctement ? Si oui, quelles sont les stratégies mises en place pour pallier à ce problème (jardin, échanges, récupération…) ?

3 Représentations autour du VIH et du VHB

3.1 Avez-vous déjà entendu parler de l'hépatite ? Que savez-vous à ce propos ?

Nous allons maintenant parler des infections par le VIH et le virus de l'hépatite B.
3.2 Selon vous, d'où proviennent ces maladies ? Comment sont-elles apparues ?
VIH :
Hépatite B chronique :

2.2 Comment peut-on les attraper ?
VIH :
Hépatite B chronique :

3.3 Selon vous, quelle partie du corps touchent-elles en particulier ?
VIH :
VHB :

3.4 En cas de contamination (ou si vous êtes concernés) est-il selon vous préférable d'aller à l'hôpital/voir un médecin généraliste/prendre des remèdes, ou plusieurs solutions à la fois ? (noter dans quel sens)

3.5 Le cas échéant, comment s'est déroulée en quoi consiste, le traitement « traditionnel » auquel vous avez recours ?

3.6 Selon vous, quel est la meilleure façon de se prémunir contre ces maladies ? Pourquoi ?

3.7 Souhaitez-vous ajouter quelque chose ?
