# Supplementary material for: Sexual Health Determinants During the Life Course and Migration of Haitian-Origin People in French Guiana: Protocol for the Parcours d’Haïti Biographical and Transdisciplinary Study
Source: JMIR Res Protoc. 2025 Jun 12;14:e63586. doi: 10.2196/63586 (PMC12203027; doi:10.2196/63586)
Supplement: Multimedia Appendix 3 [file resprot_v14i1e63586_app3.pdf]

Année de départ d'Haïti :

|_|_|_|_|

Année d'arrivée en Guyane :

|_|_|_|_|

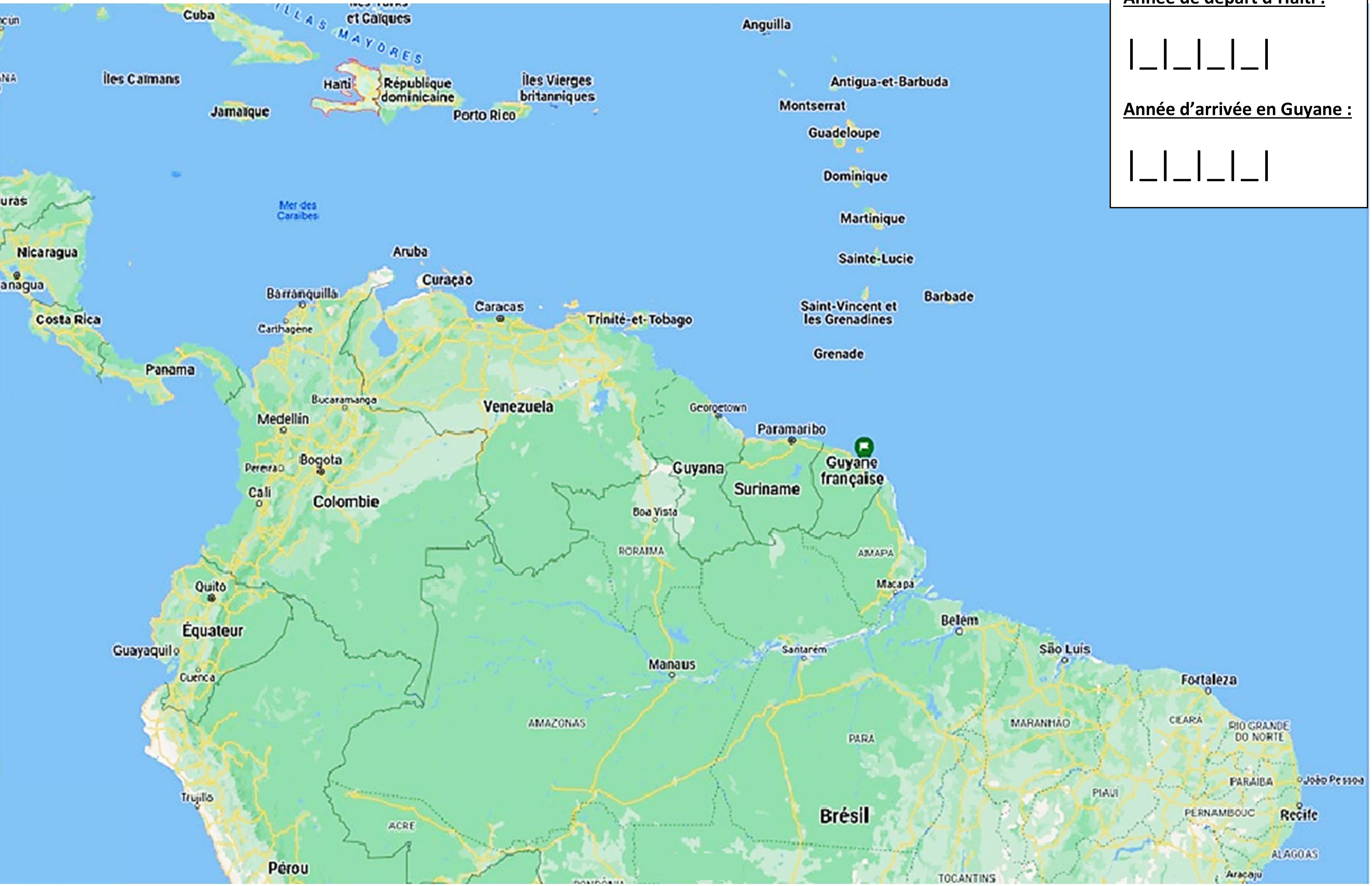

ParcoursTrajetCRF_v1_20220901

Numéro d'anonymat : |_|_|_|_| |_|_|_| |_|_| |_|
